# Drone hyperspectral imaging and artificial intelligence for monitoring moss and lichen in Antarctica

**DOI:** 10.1038/s41598-025-11535-4

**Published:** 2025-07-26

**Authors:** Juan Sandino, Johan Barthelemy, Ashray Doshi, Krystal Randall, Sharon A. Robinson, Barbara Bollard, Felipe Gonzalez

**Affiliations:** 1 QUT Centre for Robotics (QCR), Queensland University of Technology (QUT), 2 George St, 4000 Brisbane City, QLD Australia; 2 Securing Antarctica’s Environmental Future (SAEF), Queensland University of Technology (QUT), 2 George St, 4000 Brisbane City, QLD Australia; 3 Securing Antarctica’s Environmental Future (SAEF), University of Wollongong, Northfields Ave, 2522 Wollongong, NSW Australia; 4 NVIDIA, Santa Clara, CA 95051 USA; 5 SaiDynamics, Gold Coast, QLD 4211 Australia; 6 Environmental Futures, University of Wollongong, Northfields Ave, 2522 Wollongong, NSW Australia

**Keywords:** Antarctic specially protected area (ASPA), Bryophyte, Ecological monitoring, Machine learning, Semantic segmentation, Uncrewed aerial vehicle (UAV), Environmental sciences, Aerospace engineering, Electrical and electronic engineering, Software

## Abstract

Uncrewed aerial vehicles (UAVs) have become essential for remote sensing in extreme environments like Antarctica, but detecting moss and lichen using conventional red, green, blue (RGB) and multispectral sensors remains challenging. This study investigates the potential of hyperspectral imaging (HSI) for mapping cryptogamic vegetation and presents a workflow combining UAVs, ground observations, and machine learning (ML) classifiers. Data collected during a 2023 summer expedition to Antarctic Specially Protected Area 135, East Antarctica, were used to evaluate 12 configurations derived from five ML models, including gradient boosting (XGBoost, CatBoost) and convolutional neural networks (CNNs) (G2C-Conv2D, G2C-Conv3D, and UNet), tested with full and light input feature sets. The results show that common indices like normalised difference vegetation index (NDVI) are inadequate for moss and lichen detection, while novel spectral indices are more effective. Full models achieved high performance, with CatBoost and UNet reaching 98.3% and 99.7% weighted average accuracy, respectively. Light models using eight key wavelengths (i.e., 404, 480, 560, 655, 678, 740, 888, and 920 nm) performed well, with CatBoost at 95.5% and UNet at 99.8%, demonstrating suitability for preliminary monitoring of moss health and lichen. These findings underscore the importance of key spectral bands for large-scale HSI monitoring using UAVs and satellites in Antarctica, especially in geographic regions with limited spectral range.

## Introduction

Antarctica’s terrestrial ecosystems are home to freeze-tolerant vegetation like mosses and lichens, which play a crucial role in biogeochemical cycles, soil insulation, and supporting biodiversity. These organisms underpin the continent’s fragile ecosystems, increasingly threatened by climate change, extreme events, and human activities^1–15^. Monitoring Antarctic vegetation remains challenging due to harsh conditions, remoteness, limited access, and climate variability^4,5,16,17^. Traditional field surveys are time-consuming, costly, and risk disturbing sensitive vegetation^18^. While satellite imagery enables large-scale observations, its limited spectral and spatial resolution, alongside cloud interference, constrains fine-scale vegetation analysis^14,16,19–21^. Despite advances in large-scale mapping efforts, including increases in vegetation from ice retreat and moss health decline^22,23^, capturing detailed vegetation dynamics remains difficult.

Uncrewed aerial vehicles (UAVs) and global navigation satellite system (GNSS) technologies have revolutionised precise mapping in polar regions^19,21,24–27^. UAVs enable a flexible platform for deploying hyperspectral imaging (HSI) sensors and offer high-resolution data collection, while GNSS enhanced with real-time kinematic (RTK) ensures accurate geolocation for reliable vegetation analysis^28^. HSI captures a broad wavelength range, enabling discrimination of vegetation by their spectral signatures^29–32^. Together, these technologies create a powerful synergy, offering comprehensive insights into environmental dynamics and supporting biodiversity conservation, agriculture, forestry, and land use management, with minimal disturbance to sensitive habitats.

Recent advancements in Antarctic environmental monitoring have further explored multispectral imaging (MSI) data, such as that from Sentinel-2^33^. Key approaches integrating multiscale data, machine learning (ML), and UAV-based HSI sensors generate high-resolution imagery enriched with spectral details^29,34,34–37^. Each technology contributes uniquely: GNSS RTK provides georeferencing; ML techniques enable precise segmentation; and UAVs offer flexible spatial coverage and high-resolution datasets. However, unless these elements are integrated, mapping accuracy diminishes^38,39^. Moreover, limited validation of spectral libraries and simulated imagery against field data restricts the reliability of remote sensing outcomes^34,35^.

ML techniques using HSI and MSI accurately classify vegetation types and assess health^28,29,31,40,41^. However, comparisons among ML algorithms and validation of spectral indices for detecting moss health and lichen species are still limited^42,43^. Indices such as the normalised difference moisture index (NDMI), green red vegetation index (GRVI), and tasselled cap greenness (TCG) show promise in Antarctic vegetation mapping, especially for moisture differentiation and reducing misclassification from bright backgrounds^22,23^. Novel spectral indices are essential for classification tasks, particularly in polar environments where bright backgrounds impact the traditional normalised difference vegetation index (NDVI) performance. Limitations in reliable data and verification methods continue to challenge model performance assessment, requiring broader data collection^14^. Standardised workflows, validated spectral libraries, and novel spectral indices are necessary for improved moss health and lichen detection^16,19,21,31^.

**Fig. 1:**
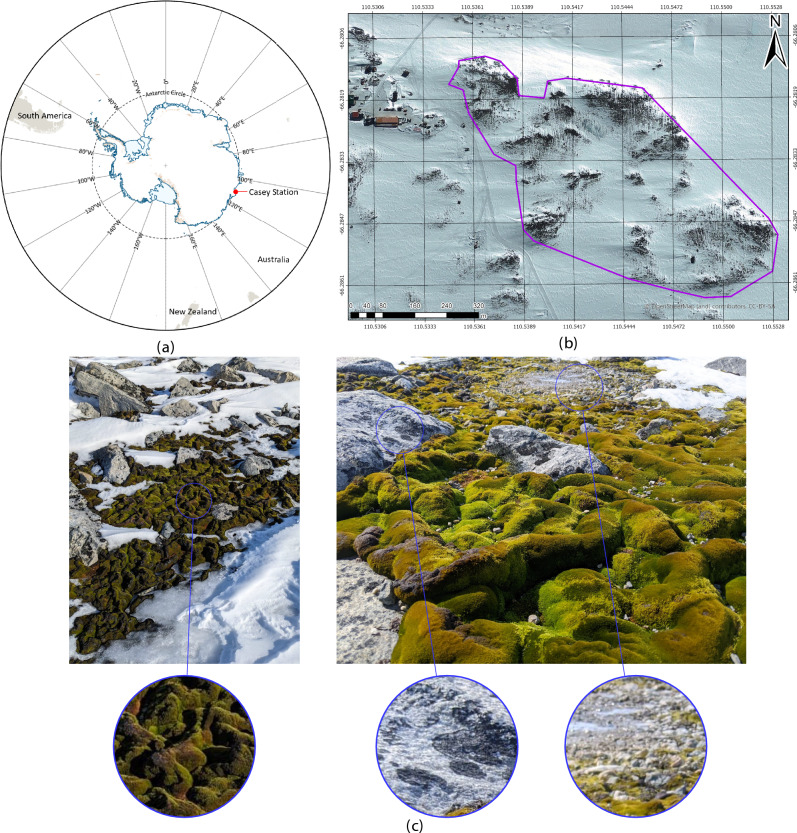
Location of ASPA 135 (66$^{\circ }$16’60” S, 110$^{\circ }$32’60” E) and studied vegetation. **(a)** Map of Antarctica showing Casey Station’s location using the Polar Stereographic Projection. **(b)** Map delineating ASPA 135 (purple) near Casey Station (top left). **(c)** Ground-level imagery of moss and lichen at ASPA 135, along with surrounding rock and ice formations.

This study addresses current gaps by building on the UAV-based HSI workflow developed by Sandino et al.^28^, which incorporated ground-based HSI data and MSI. We expand this approach by integrating UAV-captured HSI data to enhance remote sensing capabilities in polar environments. The updated methodology combines UAVs, high-resolution red, green, blue (RGB) imagery, and ground and aerial HSI data with ML-based semantic segmentation. A set of 12 configurations derived from five ML models, categorised into gradient boosting (XGBoost and CatBoost) and convolutional neural networks (CNNs) (G2C-Conv2D, G2C-Conv3D, and UNet), was tested on full and light feature sets. Full models incorporated principal component analysis (PCA) components, spectral indices, and statistical features, while light models utilised only spectral indices from eight key wavelength bands. Gradient boosting models further assessed the relevance of commonly used vegetation indices for detecting moss and lichen, vegetation which differ in size and structure to other types of vegetation. This workflow was evaluated in Antarctic specially protected area (ASPA) 135^44^, Windmill Islands, East Antarctica, focusing on lichen detection and moss health mapping (Fig. 1).

The key contributions of this study are:

1. Validating and enhancing existing workflows^28^ for processing UAV-based HSI data by incorporating novel spectral indices to improve the classification accuracy of moss and lichen.
2. Integrating data fusion and labelling techniques for precise vegetation mapping with high spatial resolution, highlighting trends in airborne hyperspectral data.
3. Providing a comprehensive comparative analysis of 12 configurations derived from five ML models (XGBoost, CatBoost, G2C-Conv2D, G2C-Conv3D, and UNet), focusing on classification performance for Antarctic vegetation monitoring.
4. Demonstrating the performance of full and light model versions, with full models (CatBoost at 98.3%, UNet at 99.7%) achieving higher accuracy, while light models (CatBoost at 95.5%, UNet at 99.8%) using only eight key wavelength bands also enabled accurate preliminary detection of moss health and lichen.
5. Offering practical insights for real-world monitoring, showing that light models with reduced spectral range are effective for preliminary vegetation mapping in polar environments where full HSI data may not always be available, facilitating rapid assessments in unexplored regions.

**Fig. 2:**
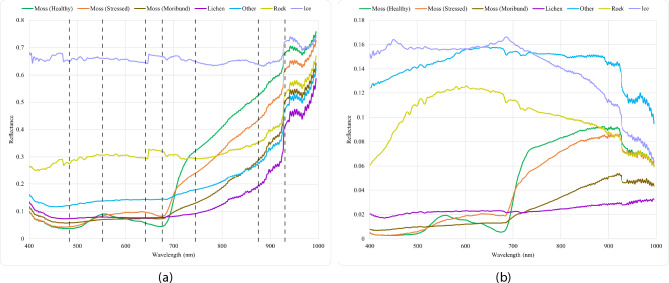
Spectral signatures of the classes under study, highlighting distinct reflection patterns. **(a)** Mean reflectance curves per class. **(b)** Standard deviation of reflectance curves per class. The spectral signatures confirm the presence of pronounced peaks and troughs at bands 480, 560, 655, 678, 740, 888, and 920 nm, consistent with previous findings^28^.

**Fig. 3:**
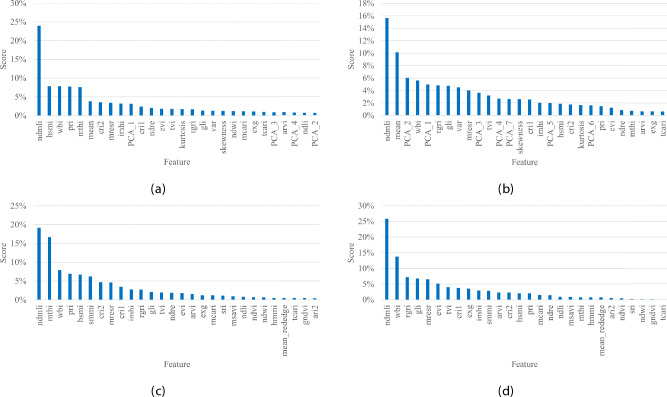
Feature importance ranking for the evaluated models: (**a**) XGB_F, (**b**) CAT_F, (**c**) XGB_L, and (**d**) CAT_L. The custom indices NDMLI, HSMI, and MTHI, presented in^28^, consistently rank highly across all models.

**Fig. 4:**
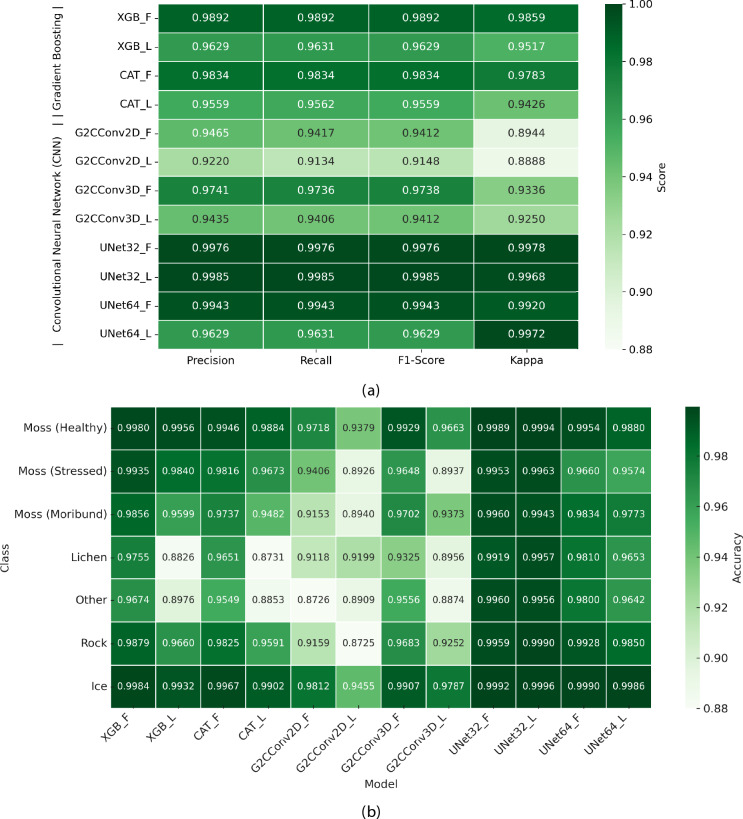
Performance summary of the tested gradient boosting and CNN models for semantic segmentation of Antarctic vegetation. (**a**) Weighted average precision, recall, F1-score, and Kappa values for mapping moss health, lichen, and other features across all tested models [see Table 5 for details]. XGBoost and UNet32 models achieved the highest Kappa values, with 98.59% and 99.78%, respectively. (**b**) F1-score values for each vegetation class, showing the highest accuracy for “Moss (Healthy)” and “Ice”, while “Lichen” and “Other” classes remain the most challenging to accurately segment.

**Fig. 5:**
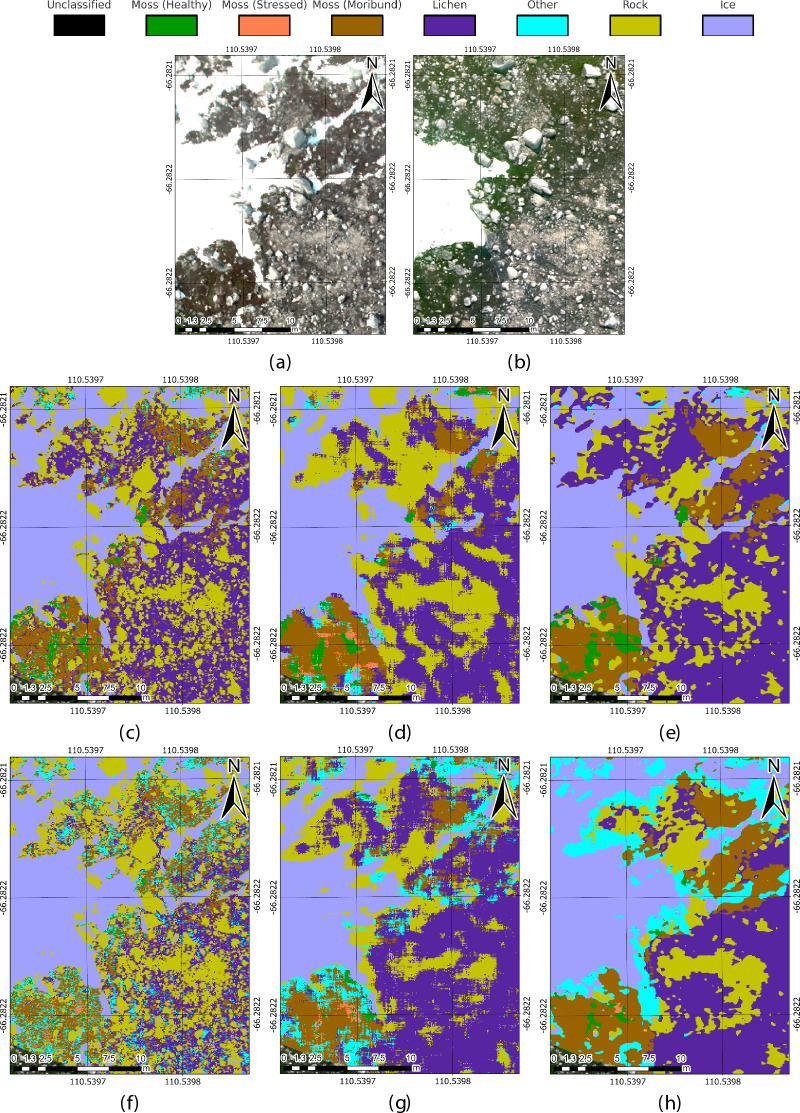
Comparison of predictions across ASPA 135 by CatBoost, G2C-Conv2D, and UNet32 models. (**a**) HSI transect at 30 m AGL. (**b**) Aligned RGB mosaic. (**c, f**) CAT_F and CAT_L variants, with full version achieving fine-grain accuracy and light version showing more noise. (**d, g**) G2CConv2D_F and G2CConv2D_L, with the full version displaying broader generalisation and the light version misclassifying “Lichen” and “Other”. (**e, h**) UNet32_F and UNet32_L, balancing detail and generalisation but with slight misclassifications in shaded areas.

**Fig. 6:**
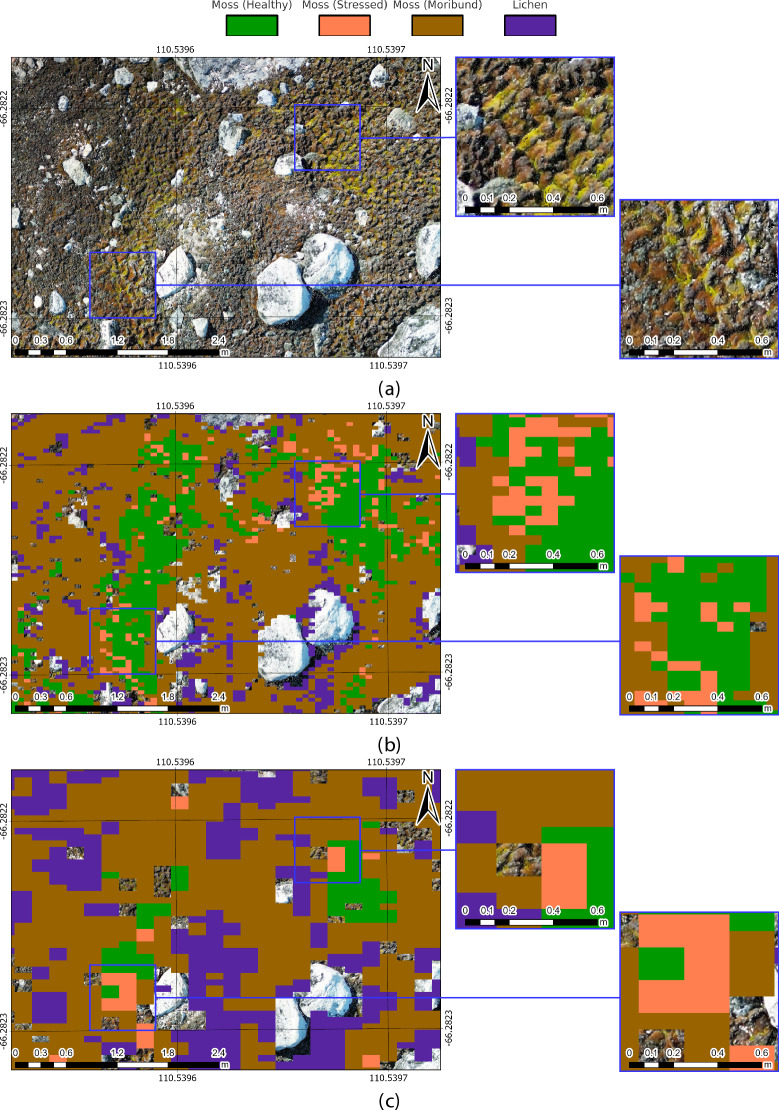
Effect of HSI resolution on moss detection at different altitudes. (**a**) RGB map from DJI Mini 3 Pro showing the surveyed area at 0.25 cm/pixel GSD. (**b**) CAT_F segmentation at 30 m AGL. (**c**) CAT_F segmentation at 70 m AGL. Higher surveying altitudes capture more extensive “Lichen” and “Moss (Moribund)” classes, though less spatial detail is obtained on healthy moss in valleys than on ridges.

**Fig. 7:**
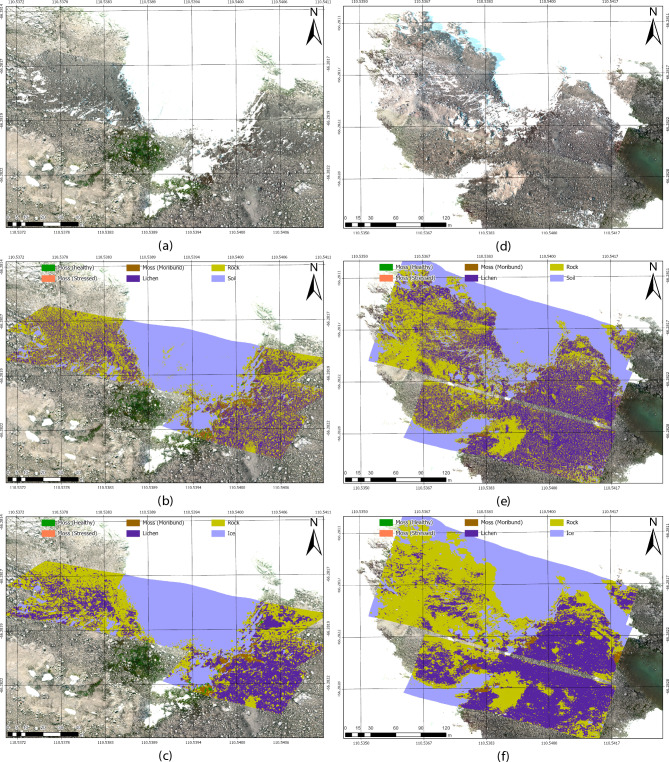
Prediction maps from hyperspectral transects collected at 30 m (**a–c**) and 70 m (**d-f**) AGL in ASPA 135. (**a**) and (**d**): RGB preview of HSI transects overlaid on the georeferenced RGB orthomosaic. (**b**) and (**e**): CAT_F model segmentation results. (**c**) and (**f**): UNet32_F model segmentation results.

**Fig. 8:**
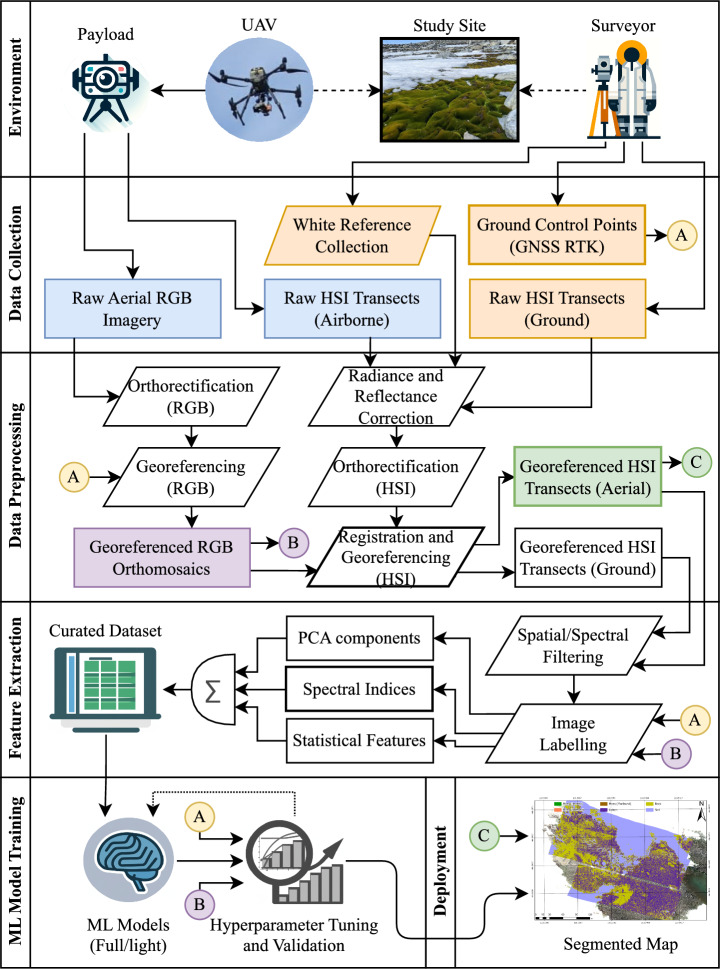
Workflow for processing UAV-acquired HSI data, enhanced with GNSS RTK ground control points and registered with high-resolution RGB orthomosaics. This approach ensures precise geolocation and pixel-wise ground truthing, enabling accurate mapping of moss health, lichen, rock, and ice.

**Fig. 9:**
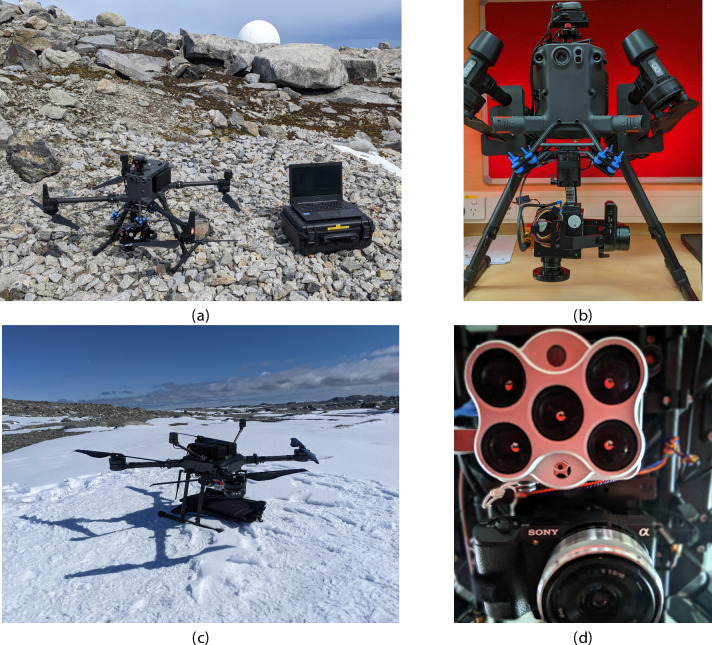
UAV platforms for hyperspectral and RGB data collection at ASPA 135. (**a**) DJI M300 RTK UAV with ground control station used for hyperspectral data collection. (**b**) Close-up of the Headwall Nano-Hyperspec hyperspectral camera mounted on the DJI M300 RTK via a Gremsy Pixy SM gimbal. (**c**) SaiDynamics Bremer BMR4-40 UAV, designed for high-resolution RGB and multispectral data collection. (**d**) Bottom-up view of the dual-camera setup on the BMR4-40, with the MicaSense Altum multispectral camera (top) and Sony Alpha 5100 high-resolution RGB camera (bottom). Only RGB data from the BMR4-40 was utilised in this study.

**Fig. 10:**
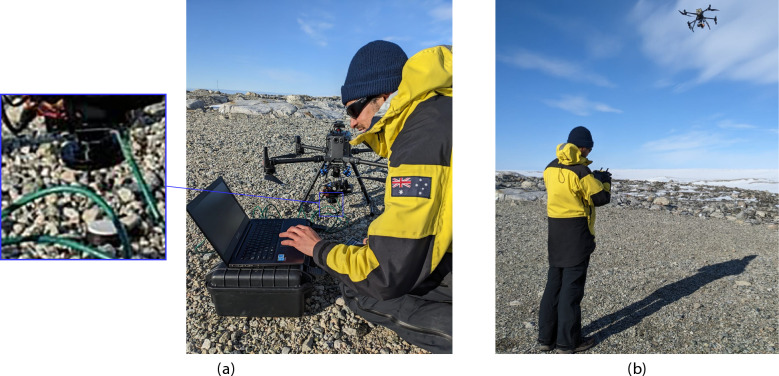
Aerial hyperspectral data collection setup in ASPA 135. (**a**) Ground setup for white reference (WR) calibration using the Nano-Hyperspec camera mounted on the DJI M300 RTK. The hyperspectral camera is directed at a Spectralon panel on the ground to calibrate exposure settings prior to UAV flights. (**b**) DJI M300 RTK equipped with the Nano-Hyperspec camera conducting flight operations over ASPA 135.

**Fig. 11:**
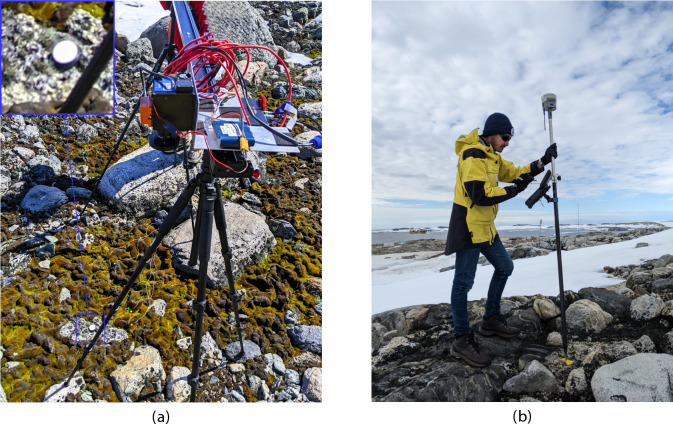
ASPA 135 ground setup for HSI and GNSS RTK data collection. (**a**) Precise positioning of a Konova slider attached to two tripods on rocks, to minimise disturbance of moss and lichen. (**b**) GNSS RTK point collection with a Trimble R10 antenna on a fixed marker with known coordinates.

**Fig. 12:**
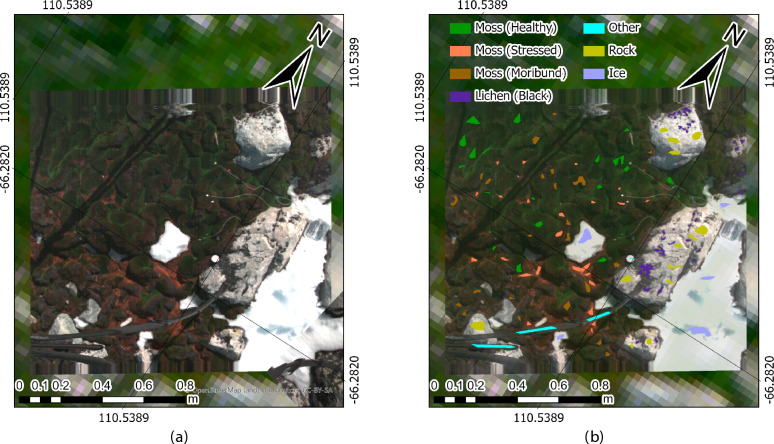
Labelling example of HSI ground data in ASPA 135. (**a**) Raw HSI scan of the ground, showing the presence of moss, lichen, the Spectralon, and non-biotic materials. (**b**) Labelling of HSI pixels per class using ENVI v5.6, where the presence of moss, lichen, and non-biotic material can be clearly identified.

**Table 1:** Validation metrics of tested ML models using ten folds of K-fold cross validation.

Model	Mean accuracy	Mean standard deviation	Mean Kappa
XGB_F	0.9894	0.0005	0.9859
XGB_L	0.9630	0.0011	0.9517
CAT_F	0.9838	0.0007	0.9783
CAT_L	0.9561	0.0007	0.9426
G2C-Conv2D_F	0.9244	0.0266	0.8944
G2C-Conv2D_L	0.8935	0.0416	0.8888
G2C-Conv3D_F	0.9450	0.0156	0.9336
G2C-Conv3D_L	0.8988	0.0477	0.9250
UNet32_F	0.9987	0.0004	0.9978
UNet32_L	0.9984	0.0006	0.9968
UNet64_F	0.9964	0.0010	0.9920
UNet64_L	0.9962	0.0024	0.9972

The gradient boosting models scored a minimum mean accuracy of 95.61% and Kappa of 94.26%, and a maximum standard deviation of 0.11%. The UNet models outperformed other models, with a minimum mean accuracy of 99.62% and Kappa of 99.20%, and a maximum standard deviation of 0.24%.

**Table 2:** Specifications of UAVs used to collect HSI and RGB data over ASPA 135.

Specification	DJI M300 RTK	Bremer BMR4-40	DJI Mini 3 Pro
Payload capacity	2.72 kg	3.7 kg	N/A
Aircraft type	Quadrotor	Quad/Octorotor	Quadrotor
Battery type	LiPo 12S	LiPo 12S	Li-ion
Weight	6.3 kg	12.0 kg	249 g
Max. Take-off Weight	9.0 kg	14.0 kg	249 g
Flight endurance	55 min	52 min	34 min
Navigation	Differential GNSS	Differential GNSS	GNSS
RTK Capable	Yes	Yes	No
Survey capacity	25 ha per flight at 70 m AGL	25 ha per flight at 70 m AGL	N/A
Operating temperature	-20$^\circ$C to 50$^\circ$C	-20$^\circ$C to 50$^\circ$C	-10$^\circ$C to 40$^\circ$ C

All UAVs were employed to collect high-quality spatial data, with the DJI M300 RTK and Bremer BMR4-40 utilised for HSI and RGB imaging, equipped with differential GNSS and RTK for precise geolocation, while the DJI Mini 3 Pro provided RGB imagery over moss beds. Here, AGL stands for above ground level.

**Table 3:** Summary of flight configurations for aerial data collection over ASPA 135.

Configuration	Date collected	AGL height	Speed	Sidelap	Overlap	GSD
		(m)	(m / s)	(%)	(%)	(cm / pixel)
M300 (HSI)	28 Jan 2023	30	1.1	15	N/a	4.6
M300 (HSI)	28 Jan 2023	70	3.0	15	N/a	10.8
BMR4-40 (RGB)	01 Feb 2023	70	3.6	80	80	2.1
Mini3Pro (RGB)	01 Feb 2023	7	1.0	70	70	0.25

Lower GSD values were targeted to achieve finer spatial resolution for moss health mapping, while higher altitude flights prioritised broader coverage. AGL denotes above ground level; GSD represents ground sampling distance.

**Table 4:** List of spectral indices used to classify moss health and lichen, totalling 28 indices.

Category	Index name	References
Vegetation	Normalised Difference Vegetation Index (NDVI)	^71^
	Green Normalised Difference Vegetation Index (GNDVI)	^72^
	Modified Soil Adjusted Vegetation Index (MSAVI)	^73^
	Enhanced Vegetation Index (EVI)	^74^
	Mean Red Edge (MRE)	^75^
	Simple Ratio Index (SRI)	^76^
	Normalised Difference Red Edge (NDRE)	^75^
	Green Leaf Index (GLI)	^77^
Chlorophyll and Pigment	Carotenoid Reflectance Index 1 (CRI1)	^78^
	Carotenoid Reflectance Index 2 (CRI2)	^78^
	Photochemical Reflectance Index (PRI)	^79^
	Red Green Ratio Index (RGRI)	^80^
	Modified Chlorophyll Absorption Ratio Index (MCARI)	^81^
Stress and Disease	Atmospherically Resistant Vegetation Index (ARVI)	^82^
	Modified Red Edge Simple Ratio (MRESR)	^83^
Water Content	Normalised Difference Water Index (NDWI)	^84^
	Water Band Index (WBI)	^85^
Other	Triangular Vegetation Index (TVI)	^86^
	Anthocyanin Reflectance Index 2 (ARI2)	^87^
	Excess Green (ExG)	^88^
	Transformed Chlorophyll Absorption Reflectance Index (TCARI)	^89^
Moss and Lichen	Stressed-Moribund Moss Index (SMMI)	^28^
	Normalised Difference Lichen Index (NDLI)	^28^
	Moss Triple Health Index (MTHI)	^28^
	Healthy-Moribund Moss Index (HMMI)	^28^
	Inverse Moss Health Index (IMHI)	^28^
	Healthy-Stressed Moss Index (HSMI)	^28^
	Normalised Difference Moss-Lichen Index (NDMLI)	^28^

The indices are categorised based on their applications in remote sensing of vegetation.

**Table 5:** List of ML models tested for vegetation mapping using airborne HSI transects.

Acronym	Description	Input features	Input layer (pixels)	Output layer (pixels)
XGB_F	XGBoost model (full feature set)	42 (PCA, indices, stats)	1 $\times$ 1	1 $\times$ 1
XGB_L	XGBoost model (light feature set)	28 (spectral indices)	1 $\times$ 1	1 $\times$ 1
CAT_F	CatBoost model (full feature set)	42 (PCA, indices, stats)	1 $\times$ 1	1 $\times$ 1
CAT_L	CatBoost model (light feature set)	28 (spectral indices)	1 $\times$ 1	1 $\times$ 1
G2CConv2D_F	G2C-Conv2D model (full feature set)	42 (PCA, indices, stats)	32 $\times$ 32	1 $\times$ 1
G2CConv2D_L	G2C-Conv2D model (light feature set)	28 (spectral indices)	32 $\times$ 32	1 $\times$ 1
G2CConv3D_F	G2C-Conv3D model (full feature set)	42 (PCA, indices, stats)	32 $\times$ 32	1 $\times$ 1
G2CConv3D_L	G2C-Conv3D model (light feature set)	28 (spectral indices)	32 $\times$ 32	1 $\times$ 1
UNet32_F	UNet model (full feature set)	42 (PCA, indices, stats)	32 $\times$ 32	32 $\times$ 32
UNet32_L	UNet model (light feature set)	28 (spectral indices)	32 $\times$ 32	32 $\times$ 32
UNet64_F	UNet model (full feature set)	42 (PCA, indices, stats)	64 $\times$ 64	64 $\times$ 64
UNet64_L	UNet model (light feature set)	28 (spectral indices)	64 $\times$ 64	64 $\times$ 64

This summary includes gradient boosting and CNN models tested with full and light feature sets to evaluate performance in segmenting Antarctic vegetation classes.

## Results

This section evaluates the spectral response of moss health and lichen, identifies key wavelengths, ranks features based on the gradient boosting models, compares the performance of the tested ML models, and assesses the prediction capabilities of the best models against unseen data.

### Spectral response of moss and lichen

The average spectral signatures, or “fingerprints”, for each class were derived based on the geometric shapes delineated during the labelling process. The mean reflectance curves for each class, along with their associated standard deviation, are shown in Fig. 2a and b, respectively.

The spectral signatures confirm the presence of pronounced peaks and troughs at wavelength bands 480, 560, 655, 678, 740, 888, and 920 nm, consistent with previous findings^28^. In contrast, non-vegetated classes like rock and ice show flatter reflectance curves, with minimal peaks, except at 660 nm, where slight variation is observed. The standard deviation of reflectance curves per class (Fig. 2b) highlights that lichen, moss (healthy), and moss (stressed) exhibit the greatest variability, indicating a higher degree of heterogeneity in their reflectance patterns. Moss vegetation classes tend to follow similar spectral trends, with most variability above 700 nm, whereas lichens present a higher deviation, and greater variability, across the visible spectrum. Non-vegetated materials such as rock and ice also show high standard deviation, likely due to the diversity of materials within these classes exhibiting different spectral properties.

### Feature ranking

The gradient boosting models (XGBoost and CatBoost) incorporate a feature ranking mechanism that allows an analysis of the relative importance of the input features in predicting the vegetation classes. Figure 3 shows the sorted ranking of feature importance for the models, revealing which features had the greatest impact on the models’ outputs.

The rankings highlight the dominant influence of the custom indices NDMLI, HSMI, and MTHI^28^, which consistently rank as the most important features for all models. Despite minor differences between the XGB_F and CAT_F models, NDMLI ranks the highest across the board. Interestingly, NDVI, a widely used vegetation index, ranked low across all models, while WBI, relevant for water content detection, ranked higher due to the inclusion of the “Ice” class. XGBoost models emphasise the importance of the PRI and CRI2 indices, while CatBoost prioritises RGRI and GLI. Full models, especially CAT_F, also place importance on statistical features like the “mean” and PCA components, though these are less critical than custom indices for moss and lichen classification. These results confirm that the models are effectively capturing the unique spectral characteristics of Antarctic vegetation, and that the custom indices are critical for accurate moss health and lichen detection.

### Performance of tested ML models

The performance of the models is evaluated using several metrics, including: (1) weighted average precision, recall, F1-score, and Kappa values; (2) intra-class F1-score mean accuracies; and (3) k-fold cross validation. Figure 4a presents a summary of the weighted average values for each model, calculated by averaging each metric across all classes, with weights assigned based on the number of true instances per class.

The performance analysis of the 12 model configurations reveals clear trends. Among the gradient boosting models, XGB_F outperforms CAT_F, achieving an F1-score of 98.92% compared to 98.34%, and a Kappa of 98.59% versus 97.83%. In both cases, the full models surpass the light versions, with XGB_L and CAT_L recording lower F1-scores of 96.29% and 95.59%, respectively. This pattern holds across all precision and recall metrics, with minor variations. For the CNN models, the UNet32 architecture is the best performer, with UNet32_L scoring 99.85% for precision, recall, and F1-score, alongside a Kappa value of 99.68%. UNet32_F follows closely, delivering similarly high scores but with a slightly higher Kappa value of 99.78%. The G2C-Conv models fall behind, with G2C-Conv3D_F performing the best in this group, reaching an F1-score of 97.38% and a Kappa value of 93.36%.

Differences between precision and recall values are minimal across all models, indicating balanced prediction capabilities. However, variations between F1-scores and Kappa values are more prominent in the CNN models, where Kappa better captures the agreement between predictions and ground truth, especially for class imbalances. A detailed analysis of model performance per class, depicted in Fig. 4b, shows that “Moss (Healthy)” and “Ice” are the easiest classes for segmentation, with UNet32_L performing best, achieving F1-scores of 99.94% and 99.96%, respectively. XGB_F and CAT_F also perform well in these classes, with XGB_F reaching 99.80% for “Moss (Healthy)” and 99.84% for “Ice”. However, classes like “Lichen” and “Other” prove more difficult, especially for light versions of the gradient boosting and G2C-Conv models. XGB_L and CAT_L record lower F1-scores of 88.26% and 87.31% for “Lichen”, and 89.76% and 88.53% for “Other”.

Among the model architectures, gradient boosting models (see Supplementary Figure S2 a for a visual model comparison) consistently perform well, with XGB_F and CAT_F achieving competitive F1-scores across most classes. Full model versions consistently outperform their light counterparts, particularly in challenging classes like “Lichen”. G2C-Conv models (Fig. S2 b) show variation, with G2C-Conv3D generally outperforming G2C-Conv2D. UNet CNNs (Fig. S2 c), especially UNet32_L, exhibit the highest F1-scores across all classes, reaffirming their superior performance. These trends, supported with consistent training and validation curves (Fig. S3 ), align with the Kappa values for UNet32 in Fig. 4a, indicating the highest agreement between predictions and ground truth, particularly for imbalanced classes. The observed performance differences between UNet32 and UNet64 may be partly attributed to the reduced number of labelled samples available when using 64 $\times$ 64 patches. Given the overlap strategy (87.5%) and patch filtering based on labelled pixels, larger patches resulted in fewer extracted samples, limiting training diversity. They also tend to be dominated by majority classes such as rock or ice, thereby exacerbating class imbalance and limiting the model’s sensitivity to smaller or rarer vegetation features.

Model accuracy was validated using a K-fold cross-validation algorithm with 10 folds, ensuring a thorough evaluation of performance across various data subsets. The validation metrics, summarised in Table 1, indicate consistent accuracy across different folds, with the gradient boosting models achieving a minimum mean accuracy of 95.61% and Kappa of 94.26%, and a maximum standard deviation of 0.11%. The UNet models outperformed other models, with a minimum mean accuracy of 99.62% and Kappa of 99.20%, and a maximum standard deviation of 0.24%. The light variants of the models demonstrated a slight drop in accuracy, with a maximum decrease of 2.77%, yet remained highly effective. This consistency in accuracy highlights the robustness of the gradient boosting and UNet models, indicating they are not overly sensitive to the specific data used for training. Additionally, the low standard deviation reinforces the stability and reliability of these models, making them strong candidates for classifying vegetation classes and other features.

### Prediction maps

To evaluate the models’ generalisation to unseen data, predictions of vegetation classes were made using the 6.5 ha aerial HSI transects from ASPA 135. While performance metrics quantify accuracy, visual assessments provide critical insights into practical applicability. Figure 5 summarises the differences observed across the best-performing models (CatBoost, G2C-Conv2D, and UNet32) when applied to aerial data. Supplementary Fig. S4 provides a comparison between the ground truth labels and the predictions from the full variants of the models on a representative labelled HSI ground scan, offering additional insight into model performance. Supplementary Figs. S5 to S7 present full visual comparisons across all models.

The gradient boosting models, particularly CAT_F (Fig. 5c), demonstrated fine-grain segmentation, particularly in low-light regions. The light variants (Fig. 5f) exhibited more noise and misclassifications, particularly in shaded areas. Similarly, the G2C-Conv2D models (Fig. 5d and g) exhibited broader generalisation with more noise, while G2C-Conv3D predictions displayed large, blob-like patches rather than detailed segmentations. UNet32 models, especially UNet32_F, showed balanced predictions, effectively capturing both fine-scale segmentation and broader patterns. However, UNet32_L and UNet64_L showed some misclassifications in the “Lichen” and “Other” classes, particularly in shaded regions. Overall, CatBoost and UNet32 models are recommended for further analysis due to their optimal balance between accuracy and fine-scale segmentation, with G2C-Conv models less suited for detailed mapping tasks.

This study compared HSI-derived prediction maps collected at different altitudes, underscoring the precision of the georeferencing and data alignment approaches (see Fig. 6 for a key comparison, and Supplementary Figs. S8 and S9 for further details). As altitude increases, prediction density for classes like “Lichen” and “Moss (Moribund)” rises, although lower altitude data remains more precise for denser patches. One observed trend (Fig. 6) is the reduced detection of healthy moss at higher altitudes, likely due to limited spatial data on moss beds in valleys versus on ridges. Stress signs appear to manifest on ridges earlier than in valleys^45^, as the larger surface area of the ridges better supports capture at high altitude.

The final output maps cover 6.5 ha of ASPA 135, showing predictions from the CAT_F and UNet32_F models. These maps offer a clear visualisation of vegetation distribution and class segmentation across the area. Figure 7 presents prediction outputs at 30 m and 70 m AGL, providing a broad view of model generalisation across varying flight altitudes. The UNet32_F model demonstrates stronger generalisation, with consistent segmentation between “Rock” and “Lichen” classes near transect edges and minor reduction in fine-scale detail at higher altitudes. In contrast, the CAT_F model shows a noticeable “wall effect” at transect corners, underscoring UNet32_F’s superior ability to generalise under different illumination and contrast conditions.

While light models show a minor decrease in detail, they remain effective for preliminary assessments when restricted to eight wavelengths. This makes them practical for broad analyses when full hyperspectral data is unavailable. Full models, however, provide greater accuracy and segmentation detail, proving their utility in targeted, high-resolution mapping.

## Discussion

This study establishes a remote sensing workflow for precise detection and mapping of Antarctic vegetation using UAV and ground HSI data, effectively addressing the logistical challenges and variable Antarctic conditions that significantly limit scientific field work^34,35^. The integration of GNSS RTK points, ground-based HSI data, and high-resolution RGB imagery provides substantial value in the workflow presented in this research. The RGB imagery provides a visual context for the classification results, while the ground-based HSI data acts as a reliable ground truth. The GNSS RTK points employed to georeference the RGB imagery and ground-based HSI data ensure precise alignment of classification results with their corresponding real-world locations. This approach is crucial for validating model accuracy, ensuring the reliability and precision of the generated maps, and facilitating detailed analysis of the predictions.

High-resolution UAV data enhances precise mapping and monitoring of moss health. The resolution differences in HSI data suggest that mapping areas with stressed or moribund moss may be more accurate at lower altitudes. High concentrations of moribund moss do not necessarily indicate overall health deterioration, as moribund mosses are more likely to occur at high-points in moss beds that receive direct radiation for most of the day^16^. Healthy mosses are often found in shaded, lower areas of the moss beds, which receive less direct radiation^14^, resulting in a lower reflectance signal that may be overpowered by the reflectance of moribund mosses. Due to the variability in moss bed structure over small spatial scales^14^, mapping at lower resolutions, even at 3 cm, can lead to a bias toward detecting moribund mosses. Coarser spatial resolutions, such as those from satellite data, compound these issues, leading to underestimation of patchy vegetation classes on the continent^22^. For optimal accuracy, continuous monitoring of these areas using the highest practicable UAV HSI resolution is advisable. Operating and collecting HSI at 30 m AGL, with a GSD of 4.8 cm/pixel, is the maximum recommended GSD for Antarctic vegetation mapping. These findings highlight the critical role of drones for environmental remote sensing in polar science. With future advancements in HSI cameras offering higher spatial resolutions, UAVs will be able to fly at higher altitudes for more precise mapping.

The algorithm’s predictive ability is not solely responsible for model accuracy; the unique spectral signatures of the target classes also play a crucial role. The differentiation of moss and lichen indices, as proposed in^28^, significantly improves the performance of models tested in this study. These indices are highly influential, unlike the traditional NDVI index, which proved less relevant for this classification task. Model robustness and adaptability in Antarctic vegetation mapping could be further assessed by including additional classes in the labelled dataset.

The systematic application of UAVs and ML techniques in Antarctica represents a shift in vegetation health assessment. Prior studies focused on potential applications rather than on standardised systematic workflows^16,24,31,35,36,46^. These studies emphasise the need for new spectral indices and reliable validation methods that remain under standardisation. This research advances ecological monitoring by integrating UAVs, RGB and HSI data, and ML, broadening the applicability of these techniques in the unique Antarctic environment^19,26,42^. Additionally, this study supports the use of customised spectral features to distinguish between moss health classes and lichens, as shown by^28^. The findings contribute spectral indices to enhance polar vegetation monitoring.

The integration of multisensor UAV platforms allows for high-resolution, flexible data collection that can capture subtle changes in Antarctic topography and vegetation. While UAVs have been used in previous studies to gather HSI data for remote sensing of Antarctic vegetation^28,29,36,47^, this research establishes a workflow that integrates high-resolution ground HSI scans with georeferenced RGB and aerial HSI scans, enabling macro-scale vegetation analysis. However, the challenges of operating under severe wind and temperature fluctuations, particularly at high altitudes and in remote locations, continue to necessitate technological advances in UAV endurance and sensor capabilities^18^.

## Conclusions and future work

This paper presented a comprehensive remote sensing workflow for mapping Antarctic vegetation using HSI data. Validated through a field campaign at ASPA 135, the workflow achieved mean accuracies and Kappa values of at least 95.61% and 94.26% for gradient boosting models, with a maximum standard deviation of 0.11%. UNet models reached 99.62% accuracy and 99.20% Kappa, with a maximum standard deviation of 0.24%. Key spectral indices—NDMLI, HSMI, and MTHI—and specific wavelengths (404, 480, 560, 655, 678, 740, 888, 920 nm) were critical to model outputs, providing effective tools for future UAV and satellite studies. These results represent a significant advance towards standardising UAV-based HSI mapping of vegetation in Antarctica. Future work may incorporate recently developed indices, such as NDMI, GRVI, and TCG, which have shown strong potential in Antarctic vegetation studies for enhancing the detection of hydrated versus dehydrated moss and minimising classification errors from bright non-vegetative backgrounds^22,23^. Leveraging these indices could further refine the spectral data processing pipeline, offering improved segmentation and monitoring capabilities for fragile Antarctic ecosystems.

In a comparative analysis of the models, gradient boosting models showed more detailed, fine-scale segmentation. XGBoost slightly outperformed CatBoost in precision, recall, and F1-scores, though CatBoost proved better at segmenting shaded areas when predicting over unseen data. G2C-Conv models offered broader predictions with more noise, making them suitable for coarser segmentation tasks. UNet32 achieved the best balance between detail and generalisation, especially at higher altitudes. Full models provided more accurate and detailed predictions, while light models were effective for preliminary assessments. Higher spatial resolution HSI datasets consistently improved accuracy, highlighting the value of high-resolution imagery for continuous monitoring of Antarctic moss beds.

Despite previous challenges in mapping lichen, this study demonstrated that the proposed spectral indices and models effectively distinguished lichen from other vegetation. UNet models, particularly UNet32, showed strong performance and should be further explored for detecting lichen classes. Future work should explore smaller patch sizes tailored to specific model architectures—particularly for G2C-Conv models—where performance could benefit from more compact spatially consistent input. Another suggested implementation is on hybrid architectures, such as UNet models incorporating G2C-Conv layers, to assess whether combining spatial-spectral sensitivity with encoder-decoder structures yields improved segmentation performance. Exploring the performance of CNNs trained directly on raw spectral bands, and performing band selection to identify the minimum number of wavelengths required for reliable classification, is another valuable direction for reducing preprocessing complexity and better leveraging model capacity. Evaluating UAV MSI data is also recommended to validate the use of simpler models in large-scale Antarctic vegetation mapping. Given the limited availability of labelled UAV HSI data, semi-supervised methods such as SAM^48^ and unsupervised models such as GC-ViT^49^ offer promising alternatives for efficient labelling and segmentation.

## Methods and materials

This research builds upon and extends the workflow for mapping vegetation in Antarctica, initially proposed by Sandino et al.^28^, by incorporating UAV-acquired HSI data. The dataset is further enhanced with GNSS RTK ground control points (GCPs) and registered with high-resolution RGB drone orthomosaics, ensuring precise geolocation and pixel-level ground truthing. The key stages of this workflow are illustrated in Fig. 8. The following sections delve into the practical implementation and refinement of this methodology, detailing the integration and analysis of airborne HSI data for accurate mapping of moss health, lichen distribution, rock, and ice coverage. Informed consent was obtained to publish images of the expeditioner, as shown in this section, in an online open access publication.

### Study site

The UAV missions surveyed ecologically significant moss bed locations in ASPA 135 (66$^{\circ }$16’60” S, 110$^{\circ }$32’60” E) on Bailey Peninsula in East Antarctica’s Windmill Islands^44^. The ASPA, as shown in Fig. 1, is adjacent to the Australian Casey Research Station, spanning an area of approximately 28 hectares (ha)^44^, and was visited on three separate occasions from January 28 to February 2, 2023. Drone imagery and GCP collection at ASPA 135 were limited to short periods between 3:30 pm and 6:30 pm (UTC +9) due to challenging weather conditions. Sky conditions ranged from partly clear to fully clear, with a mean observed temperature of – 2 $^\circ$C and average wind gusts of 5.14 m/s.

### UAV and sensing platforms

Three UAV platforms were employed for aerial data collection over the ASPA: (1) the DJI Matrice 300 RTK (DJI, Nanshan, Shenzhen, China); (2) the Bremer BMR4-40 (SaiDynamics, Gold Coast, Australia); and 3) the DJI Mini 3 Pro (DJI, Nanshan, Shenzhen, China). The DJI M300 RTK, selected for its robust performance in Antarctic conditions, was the primary platform for HSI data acquisition. The Bremer BMR4-40, a custom-built UAV supporting multiple payloads, was critical for capturing MSI and RGB data. The DJI Mini 3 Pro conducted low-altitude surveys over densely vegetated areas, providing detailed observations crucial to our study, and also served as a swift reconnaissance tool for terrain assessment. Specifications for each UAV are provided in Table 2.

The DJI M300 RTK carried a Headwall Nano-Hyperspec (Headwall Photonics, Boston, US) hyperspectral camera (Fig. 9a). This push-broom sensor captures one-dimensional (1D) scans across up to 270 spectral bands between 400 nm and 1000 nm. The camera weighs 680 g, has a 50.68$^\circ$ field of view (FOV), and offers a spatial resolution of 640 pixels. As shown in Fig. 9b, the Nano-Hyperspec was mounted on a Gremsy Pixy SM gimbal (Gremsy, Ho Chi Minh City, Vietnam) to maintain a nadir view and reduce UAV motion effects.

Figure 9c shows the BMR4-40 UAV, equipped with dual payloads: a MicaSense Altum (AgEagle, Kansas, US) MSI camera and a Sony Alpha 5100 (Sony Group Corporation, Tokyo, Japan) RGB camera (Fig. 9d). The Alpha 5100, operating with a global shutter, provides a 24.3 MP resolution and an 83$^\circ$ FOV. While MSI data was also collected, this study focuses on RGB data; detailed MSI analysis for Antarctic vegetation can be found in Raniga et al.^41^.

### Flight configurations

Four distinct flight configurations were used: two for the DJI M300 RTK, one for the BMR4-40, and one for the Mini 3 Pro. A summary of these configurations is presented in Table 3. The first M300 configuration captured high-resolution HSI data, yielding a ground sampling distance (GSD) of 4.6 cm/pixel. The second M300 configuration was for lower-resolution HSI, with a GSD of 10.8 cm/pixel. The Mini 3 Pro configuration provided very high-resolution RGB data over moss bed locations, while the BMR4-40 configuration captured data over the entire ASPA. All flight missions followed lawnmower patterns for consistent image collection. The missions were pre-planned and uploaded to the UAVs using DJI Pilot 2, DJI Fly, and Mission Planner for the M300, Mini 3 Pro, and BMR4-40, respectively.

### Hyperspectral camera setup

As the Headwall Nano Hyperspec camera operates as a push-broom scanner, the UAV’s ground speed calculations depend on field illumination conditions prior to each flight. Ground speed estimation was achieved using the Hyperspec III *FOV Estimator* tool (Headwall Photonics, Boston, US), which calculates the optimal speed by factoring in both the camera’s exposure time and the mission’s above ground level (AGL) height. A white reference (WR) scan is essential for setting the camera’s optimal exposure, captured in-field before each flight.

To determine this exposure, the hyperspectral camera was positioned to scan a white 99% reflective Spectralon panel (Labsphere, North Sutton, US), as illustrated in Fig. 10, placed at the drone’s takeoff point. The Hyperspec III software was used to adjust the exposure time, targeting approximately 80% spectral gain when the camera cursor was focused on the Spectralon panel.

Once the exposure time was set, the FOV calculator estimated the necessary UAV ground speed for the mission, programmed according to the parameters in Table 3. To maintain data accuracy, WR scans were captured before and after each flight mission to calibrate reflectance consistently.

### Ground surveys

Ground surveys were conducted to gather essential ground truth data and validate the aerial HSI and RGB imagery. This data collection involved two primary tasks: 1) obtaining ground-based HSI scans over moss beds and lichen; and 2) collecting GNSS RTK points from physical markers, also known as ground control points (GCPs).

Due to limitations in the spatial resolution of aerial HSI transects for interpreting and extracting detailed textural features, ground HSI scans were essential to increase labelling accuracy. To capture HSI data over moss beds, we selected sites ($n = 11$) based on their significance for ongoing monitoring of moss health and community composition in the ASPA^5,16,50^. As previously described^28^, these 11 HSI scans were captured from heights under 2 m AGL using a Konova slider (Konova, Daejeon, South Korea) mounted on tripods, covering approximately 1.2 m by 1.35 m per scan (see Fig. 11a for details). The camera and slider speed setup followed the procedure used for aerial HSI data, with WR scans embedded within each ground-based scan.

For GNSS RTK point collection, a Trimble R10 antenna was employed, as illustrated in Fig. 11b. The RTK system consists of a Trimble R10 base antenna at a fixed marker with known GNSS coordinates and a rover antenna that receives live GNSS corrections. A continuous static survey of at least six hours was conducted to achieve high-confidence GNSS coordinates for the base marker. The GNSS survey required a minimum of two days to complete. In total, 44 GNSS RTK points were gathered, covering nine GCPs and 35 vegetation-focused locations. Post-processing of the GNSS logs was performed using the AUSPOS GNSS service from Geoscience Australia to finalise the GNSS RTK point dataset^51^.

### Data preprocessing

Processing UAV imagery for vegetation detection and classification requires creating georeferenced maps from individual images and scans. This workflow, as outlined in the overall structure, includes calibration for reflectance, followed by orthorectification and georeferencing.

Orthorectification and georeferencing of RGB data from Sony and Mini 3 Pro cameras were completed using Agisoft Metashape v2.1.0 (Agisoft LLC, Petersburg, Russia) and ArcGIS Pro v3.2 (ESRI, Redlands, CA, US), respectively. The RGB orthomosaics, crucial for HSI data alignment, showed precise error metrics: longitudinal (X) error at 0.80 m, latitudinal (Y) error at 0.50 m, and altitude (Z) error at 1.08 m, yielding a combined XY error of 0.94 m and a total positional error of about 1.43 m. RGB georeferencing used a second-order polynomial transformation with nine GNSS RTK points for enhanced accuracy. Root-mean-square (RMS) error metrics validated the georeferencing quality, revealing minimal errors in forward, inverse, and combined forward-inverse transformations ($5.45 \times 10^{-3}$, $5.48 \times 10^{-3}$, and $3.00 \times 10^{-6}$, respectively), indicating excellent alignment precision.

HSI data was processed using SpectralView (Headwall Photonics, Boston, US) for calibration to radiance values, followed by orthorectification to adjust for sensor-induced distortions. Georeferencing was conducted in ArcGIS Pro, using the RGB orthomosaics as reference rasters. A spline technique with over 80 reference points per transect ensured precise HSI alignment, taking two to four hours per scan. Ground HSI scans were similarly aligned, employing a first-order polynomial technique with approximately six reference points per scan. Reflectance for HSI data was calculated using Python scripts within the Spectral Python library^52^, applying the following formula:

1 $$\begin{aligned} R(\lambda ) = \left( \frac{L(\lambda )}{L_{\text {ref}}(\lambda )}\right) \cdot R_{\text {ref}}(\lambda ), \end{aligned}$$

where $R(\lambda )$ is reflectance, $L(\lambda )$ is radiance, $L_{\text {ref}}(\lambda )$ is WR radiance, and $R_{\text {ref}}(\lambda )$ is WR reflectance at wavelength $\lambda$. For the 99% reflective Spectralon used here, the calculation simplifies to:

2 $$\begin{aligned} R(\lambda ) = \frac{L(\lambda )}{L_{\text {ref}}(\lambda )}. \end{aligned}$$

This process yielded 18 georeferenced orthomosaics covering 6.505 ha from HSI data, a Sony RGB map over 15.427 ha, and a Mini 3 Pro map spanning 0.4059 ha. The alignment quality of these orthomosaics and differences in ground sampling distance (GSD) are illustrated in Fig. S1, highlighting variations due to changing snowfall, which impacted ice coverage observed in aerial HSI imagery compared to other sensors. This discrepancy, due to snowfall timing, aligns with details in Table 3.

### HSI data labelling

The moss and lichen communities under study are relatively small compared to the surrounding rock and ice and are often interspersed with other materials. Due to the lower spatial resolution in aerial HSI data, annotations for supervised ML classification were made using ground HSI scans, which offer a GSD of up to 0.3 cm/pixel. This study follows and expands the labelling process established in King et al.^16^ and Sandino et al.^28^, defining the following classes:

1. Moss (Healthy): indicative of robust and healthy green moss beds.
2. Moss (Stressed): exhibiting yellow, red, or orange hues due to new pigment production in response to stress^45,53,54^.
3. Moss (Moribund): light grey, brown, or black in color, reflecting advanced health decline and loss of photosynthetic pigments.
4. Lichen: various common Antarctic lichens such as *Usnea* spp., *Umbilicaria* spp., and *Pseudephebe* spp.
5. Other: includes non-biotic items, such as plastic and metal.
6. Rock: representing exposed rock and soil surfaces.
7. Ice: capturing the ice surfaces.

HSI scans were labelled using ENVI v5.6^55^, where polygons were drawn over homogeneous areas within each class. The labelled output, illustrated in Fig. 12, shows clearly identifiable moss, lichen, and other non-biotic materials. The total count of labelled pixels for each class is provided in Table S1.

### Feature extraction

The extraction of features from HSI data included principal component analysis (PCA), spectral indices, and statistical features, each capturing critical spectral characteristics for accurate ML-based segmentation.

#### Principal component analysis (PCA)

PCA^56^ is widely applied to HSI data^57,58^ to reduce dimensionality while retaining key spectral information. With HSI data comprising hundreds of bands, PCA transforms this high-dimensional data into a smaller set of orthogonal principal components (PCs) representing maximum variance. The top 10 PCs were selected for their ability to encapsulate key spectral variations in the data, enhancing the models’ classification performance.

#### Spectral indices

The spectral data from HSI scans provides ML models with rich information for precise classification. Moss and lichen characteristics are captured by computing various spectral indices, totalling 28, which were chosen based on their ability to assess features like vegetation health, water content, chlorophyll, and stress markers. The indices, which are listed in Table 4, fall into three categories: (1) established indices commonly used across vegetation remote sensing^59^; (2) indices applied in prior Antarctic vegetation studies^16,19,29^; and (3) new indices proposed for moss health and lichen classification^28^. Recent advancements in Antarctic vegetation research have further supported the utility of indices such as NDMI, GRVI, and NDRE, which improve the detection of subtle variations in moisture and vegetation density^22^. The TCG index has also been shown to outperform NDVI in these regions, particularly when mapping moss beds and lichen communities in bright, reflective environments^23^. While our current index selection builds upon the foundation established in earlier studies, incorporating these indices in future work could further enhance classification performance.

#### Statistical features

To enrich model inputs, a set of statistical features capturing the distribution of reflectance was derived from HSI data:

- Mean: Average intensity across pixels within a class, offering a basic spectral summary.
- Variance: Measures intensity dispersion around the mean, indicating uniformity within a class.
- Skewness and Kurtosis: Represent the asymmetry (skewness) and sharpness (kurtosis) of pixel intensity distributions.

### ML model training

This study evaluated 12 state-of-the-art configurations derived from five supervised ML models for semantic segmentation, categorised into gradient boosting and CNNs. Gradient boosting models such as XGBoost^60^ and CatBoost^61^ are known for their robust performance in handling large datasets with sparse features, making them suitable for high-dimensional data like hyperspectral imagery. These models iteratively enhance prediction accuracy by minimising errors through boosting while using regularisation techniques to prevent overfitting^60,61^.

CNNs, renowned for their performance in image classification and semantic segmentation, were also evaluated, including G2C-Conv2D^62^, G2C-Conv3D^62^, and two variants of the UNet architecture^63^ with input sizes of 32 and 64 pixels each. CNNs excel at detecting spatial and spectral patterns in high-dimensional data, using convolutional layers to identify pixel relationships and fine details in hyperspectral data^64^. G2C-Conv2D and G2C-Conv3D are lightweight convolutional models designed to capture contextual differences between neighbouring features through channel-difference operations in 2D and 3D, respectively. Both models were implemented as complete classification architectures following the original design by Roy et al.^62^. In G2C-Conv2D, the input patch is treated as a 2D tensor where each channel corresponds to a spectral band; in G2C-Conv3D, the patch is treated as a volumetric cube, allowing spectral and spatial dimensions to be jointly convolved. Both architectures use three convolutional blocks with max pooling and a final fully connected layer to predict the class of the central pixel within the patch.

The UNet models follow a standard encoder-decoder structure with skip connections, enhanced in this study with Squeeze-and-Excitation (SE) blocks to model channel-wise attention. Each downsampling block includes dual convolutions with batch normalisation and ReLU activation, followed by an SE block and max pooling. Upsampling is achieved using transposed convolutions, with concatenated skip connections and dual convolutions with SE blocks. A 1 $\times$1 convolution in the final layer maps the feature maps to class probabilities across the entire patch. UNet’s symmetrical design supports patch segmentation, making it highly effective for detailed vegetation mapping^63^, as shown in recent studies for Antarctic moss and lichen segmentation^41^.

#### Model configuration

Each model was trained on two datasets:

1. A full feature set combining PCA-derived principal components, spectral indices, and statistical features.
2. A light version using only selected spectral indices.

The first configuration leverages both spectral and statistical properties of vegetation classes, while the second assesses the effectiveness of spectral indices alone, simulating scenarios with limited spectral data (e.g., satellite imagery). Further details on data and model configurations are listed in Table 5.

In the gradient boosting models, each pixel is treated individually, with 1 $\times$ 1 input and output layers, allowing for per-pixel segmentation. Conversely, G2C-Conv2D and G2C-Conv3D models classify individual pixels within patches (e.g., 32 $\times$ 32 pixels), while UNet models, capable of flexible input-output sizes, were tested with 32 $\times$ 32 and 64 $\times$ 64 patches, enabling direct comparisons of segmentation performance by patch size.

#### Data preparation

Labelled hyperspectral pixels and patches were sampled from the full dataset according to model requirements. Gradient boosting models received pixel-wise tabular data, filtering out unlabelled pixels during training. All training inputs of the CNN models (i.e., G2C-Conv2D, G2C-Conv3D, and UNet) consisted of labelled patches extracted using an 87.5% overlap to maximise coverage and spatial context.

Data filtering operations, including wavelength and spectral filtering, as well as spatial smoothing, were applied to enhance input HSI quality. Non-informative bands were removed (i.e., 428-430 nm, 754-768 nm, 932-936 nm, and 998-1002 nm) due to noise and artifacts captured by the HSI camera. Spectral filtering used a Savitzky-Golay filter with a window of 3 and polyorder of 2 to retain critical spectral details.

#### Data augmentation and preprocessing

Several preprocessing steps ensured data quality for model training. Using a fixed random seed of 32, 70% of labelled pixels or patches (depending on the model) were allocated for training, and 30% for validation (see Table S1 ). Min-max normalisation scaled features to [0, 1], enhancing convergence rates and balancing feature influence.

Data augmentation techniques were applied to improve CNN model generalisation, allowing them to better handle variations in the input data. The implemented augmentations in this study via the PyTorch library are:

- **Random Resizing:** The labelled patches were resized with random scaling and aspect ratio adjustments to simulate different object sizes and perspectives, while maintaining patch dimensions.
- **Gaussian Noise Addition:** Gaussian noise was added to the data to mimic sensor noise and improve model robustness to such artefacts.
- **Brightness Adjustment:** Pixel intensities across all hyperspectral bands within each labelled patch were uniformly scaled by a brightness factor to help the models generalise across varying illumination levels.

These augmentation strategies were selected based on empirical testing of multiple combinations, consistently yielding better segmentation performance than alternatives such as rotation, flipping, and affine transformations. These latter techniques were excluded as they degraded model performance—likely due to the lower spatial resolution of hyperspectral imagery, where geometric changes can disrupt the shape and structure of vegetation features. The optimal values for augmentation, and model hyperparameters were optimised via grid search and are detailed in Table S2 and Table S3. Although validation scores showed marginal differences across configurations (see Supplementary Table S4), visual inspection of segmentation maps highlighted noticeable degradation when using geometric augmentations, as illustrated in Supplementary Fig. S10.

#### Model training and experimental setup

Gradient boosting models used a learning rate of 0.1 and maximum depth of 6 with early stopping (10 rounds). Full hyperparameters for gradient boosting models are in Table S2 . CNNs used distinct optimisers and loss functions tailored to enhance segmentation accuracy. The G2C-Conv models employed the Adam optimiser with a learning rate (LR) of 0.001, weight decay of $1 \times 10^{-5}$, and AMSGrad enabled, using cross-entropy loss for multi-class segmentation. The UNet models were trained with RMSprop, featuring a learning rate of 0.001, momentum of 0.9, and weight decay of $1 \times 10^{-5}$. A custom loss function, combining cross-entropy (0.7), dice (0.1), and intersection over union (IoU) (0.2) losses, was used to refine segmentation precision. This combination balances pixel-wise accuracy (cross-entropy) with region overlap (IoU) and class imbalance sensitivity (dice), and yielded better results during empirical testing than any single loss alone. Further hyperparameter details are provided in Table S3 .

Models were compiled on a high-performance desktop with a 24-core Intel^®^ Core^®^ i9-13900 CPU, 64 GB RAM, NVIDIA GeForce RTX 4090, and 1 TB SSD. Python libraries (Spectral v0.23.1^52^, Pandas v2.0.3^65^, Numpy v1.25.0^66^) were used for HSI processing, while Scikit-learn v1.3.2^67^, XGBoost v2.0.2^60^, CatBoost v1.2.2^61^, and PyTorch v2.4.0^68^ powered the models.

Model evaluation metrics included precision, recall, F1-score, and Kappa. Precision gauges model predictive accuracy, recall measures identification completeness, F1-score balances precision and recall, and Kappa assesses prediction-ground truth alignment^69,70^. The metrics are computed as:

3 $$\begin{aligned} \text {Precision}&= \frac{TP}{TP + FP}, \end{aligned}$$

4 $$\begin{aligned} \text {Recall}&= \frac{TP}{TP + FN}, \end{aligned}$$

5 $$\begin{aligned} \text {F1-Score}&= \frac{2}{\frac{1}{\text {Precision}} + \frac{1}{\text {Recall}}}, \end{aligned}$$

6 $$\begin{aligned} \text {Kappa}&= \frac{P_o - P_e}{1 - P_e}, \end{aligned}$$

where *TP*, *FP*, and *FN* are true positives, false positives, and false negatives, respectively. Observed accuracy $P_o$ is model accuracy, and expected accuracy $P_e$ represents random accuracy. The random accuracy is calculated as the sum of the products of the row and column totals divided by the square of the total number of samples.

## Supplementary Information

Supplementary Information.

## Data Availability

All data generated during this study, including maps, metadata, and performance metrics, can be accessed at https://dx.doi.org/doi:10.26179/r0e3-2j72, or via the Australian Antarctic Data Centre (AADC) https://data.aad.gov.au, entry ID: AAS_4628_UAS_ASPA135_AERIAL_HSISEG_MOSSLICHEN_2023.
