# Supplementary material for: Drone hyperspectral imaging and artificial intelligence for monitoring moss and lichen in Antarctica
Source: Sci Rep. 2025 Jul 26;15:27244. doi: 10.1038/s41598-025-11535-4 (PMC12297592; doi:10.1038/s41598-025-11535-4)
Supplement: Supplementary file 1 — Supplementary Information. [file 41598_2025_11535_MOESM1_ESM.pdf]

Supplementary Information

Class	Name	Labelled Pixels
1	Moss (Healthy)	18,582
2	Moss (Stressed)	14,969
3	Moss (Moribund)	22,212
4	Lichen	31,071
5	Other	35,038
6	Rock	65,575
7	Ice	123,453
	Total	310,900

Table S1. Total count of labelled hyperspectral pixels per vegetation and material class in ASPA 135, totalling 310,900 pixels. The high count of labelled pixels for classes such as rock and ice reflects their extensive presence in the dataset, while the moss categories contain fewer labelled pixels, highlighting the challenge of capturing accurate class distributions in sparse and patchy vegetation. These labelled pixel counts form the foundational data for model training and validation in this study.

Hyperparameter	CatBoost Value	XGBoost Value
Number of iterations / trees	1,000	140
Learning rate	0.03	0.1
Maximum tree depth	5	5
Random strength (for scoring splits)	1	-
Bagging temperature	1	-
Overfitting detector type	'Iter'	Early stopping enabled
Overfitting detector patience	6	6
Maximum size for one-hot encoding	2	-
Complexity of categorical features	2	-
Task type / Use GPU	'GPU'	True
Number of parallel threads	16	16
Booster type	-	'gbtree'
Sampling method	-	'gradient_based'
Subsample ratio of the training instances	-	0.85
Subsample ratio of columns when constructing each tree	-	0.9
Minimum loss reduction required to make a split	-	0.0
L1 regularization term on weights	-	0.0
L2 regularization on leaves	5	1.0

Table S2. Comprehensive list of hyperparameters used for the gradient boosting models. The hyperparameters were carefully selected to find the right balance between model complexity, generalisation capabilities, and computational efficiency.

Hyperparameter	G2CConv Value	UNet Value
Patch size (pixels)	32	32 (UNet32), 64 (UNet64)
Patch overlap (%)	87.5	87.5
Excluded classes	[0]	[0]
Batch size	128	128
Learning rate	1×10^{-3} (2D), 1×10^{-4} (3D)	1×10^{-3}
Number of epochs	100	100
Early stopping patience	6	6
Scheduler patience	3	3
Data augmentation (enabled)	True	True
Resize parameters (scale)	[0.8, 1.0]	[0.8, 1.0]
Noise augmentation (mean, std)	(0.0, 0.05) (2D), (0.0, 0.1) (3D)	(0.0, 0.1)
Brightness factor	0.2	0.2
Gradient clipping	1.0	1.0
Scaling (mean)	True	True
Scaling (std)	True	True
PCA components	10	10
Cross-validation (k-fold)	10	10

Table S3. Comprehensive list of hyperparameters used for the CNN models. The hyperparameters were carefully selected to find the right balance between model complexity, generalisation capabilities, and computational efficiency.

Technique/Model	mean_OA	std_OA	mean_AA	std_AA	mean_Kappa	std_Kappa
none/G2C-Conv2D_32	98.638	0.111	98.273	0.085	98.218	0.146
none/G2C-Conv3D_32	98.725	0.221	98.430	0.237	98.333	0.289
none/UNet_32	99.970	0.007	99.953	0.010	99.952	0.011
none/UNet_64	99.860	0.042	99.684	0.070	99.754	0.073
brightness/G2C-Conv2D_32	98.510	0.058	98.282	0.200	98.052	0.075
brightness/G2C-Conv3D_32	98.917	0.049	98.696	0.029	98.584	0.064
brightness/UNet_32	99.956	0.010	99.941	0.009	99.930	0.016
brightness/UNet_64	99.772	0.070	99.564	0.135	99.600	0.123
noise/G2C-Conv2D_32	93.982	0.839	93.203	0.912	92.159	1.082
noise/G2C-Conv3D_32	93.644	0.951	92.808	1.066	91.708	1.225
noise/UNet_32	99.910	0.004	99.863	0.018	99.856	0.006
noise/UNet_64	99.711	0.038	99.430	0.073	99.494	0.066
resize/G2C-Conv2D_32	98.452	0.429	98.253	0.447	97.976	0.560
resize/G2C-Conv3D_32	98.626	0.229	98.374	0.233	98.204	0.299
resize/UNet_32	99.939	0.013	99.902	0.029	99.903	0.021
resize/UNet_64	99.727	0.023	99.486	0.028	99.522	0.039
flip/G2C-Conv2D_32	98.359	0.264	98.013	0.317	97.854	0.345
flip/G2C-Conv3D_32	98.720	0.170	98.470	0.119	98.325	0.222
flip/UNet_32	99.380	0.048	98.333	0.150	99.006	0.078
flip/UNet_64	97.825	0.256	95.737	0.402	96.206	0.442
affine/G2C-Conv2D_32	98.056	0.195	97.859	0.097	97.459	0.254
affine/G2C-Conv3D_32	98.725	0.025	98.496	0.049	98.333	0.032
affine/UNet_32	99.768	0.079	99.666	0.104	99.627	0.127
affine/UNet_64	99.130	0.157	98.054	0.376	98.478	0.274
rotate/G2C-Conv2D_32	98.766	0.086	98.655	0.038	98.387	0.112
rotate/G2C-Conv3D_32	98.551	0.247	98.287	0.253	98.105	0.323
rotate/UNet_32	99.925	0.010	99.882	0.009	99.879	0.015
rotate/UNet_64	99.488	0.032	99.019	0.036	99.104	0.056

Table S4. Statistical summary of the ablation study on data augmentation techniques, presenting the mean and standard deviation of average accuracy, overall accuracy, and Kappa scores after running each combination at least three times.

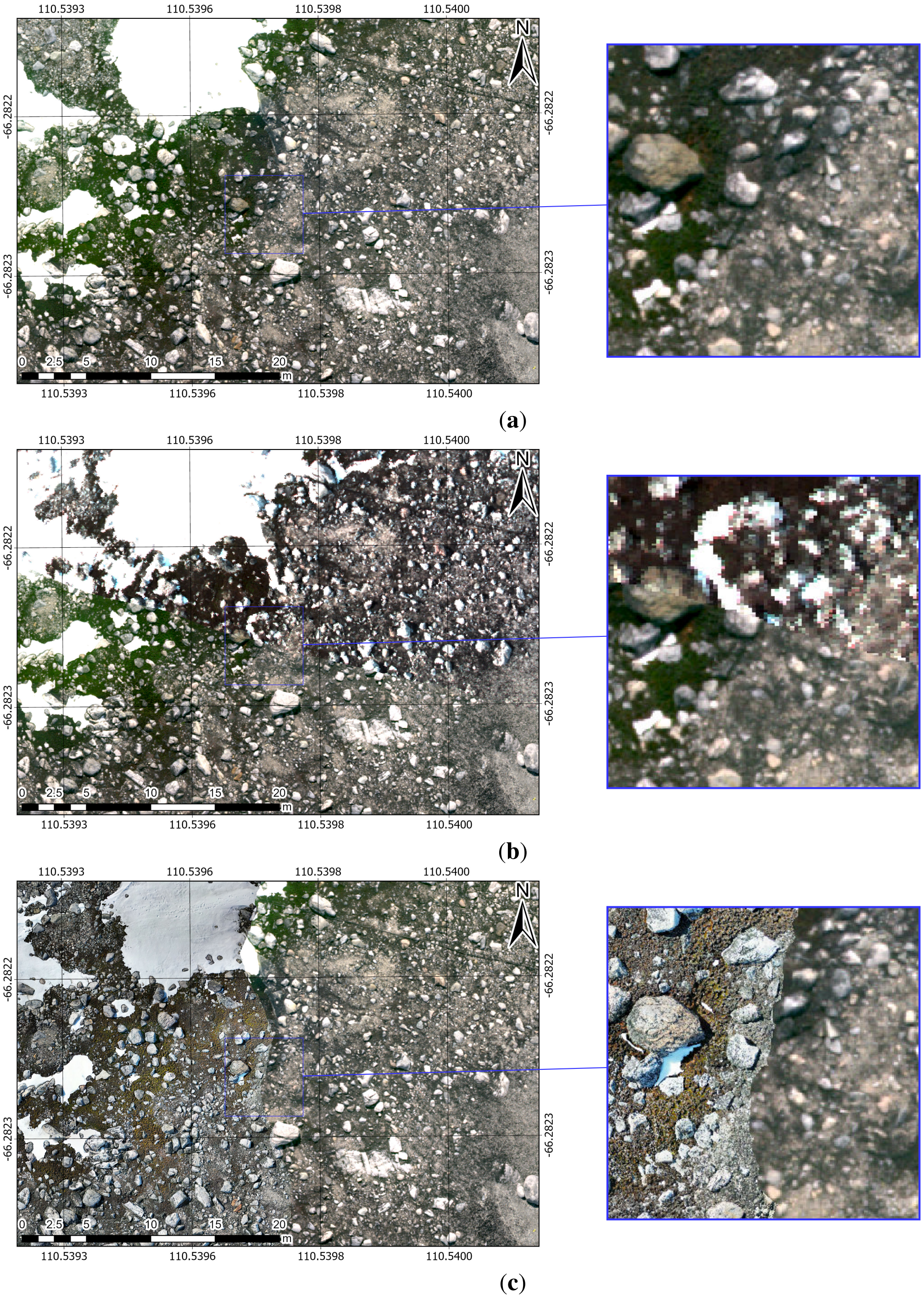

Figure S1. Georeferenced orthomosaics of RGB and HSI data over ASPA 135. (**a**) RGB map (2.0 cm / pixel GSD) providing a baseline for spatial referencing; (**b**) HSI transects (4.8 cm / pixel GSD) detailing spectral characteristics; (**c**) High-resolution Mini 3 Pro map (0.25 cm / pixel GSD) instrumental for data labelling and validation, enhancing the accuracy of the vegetation mapping process.

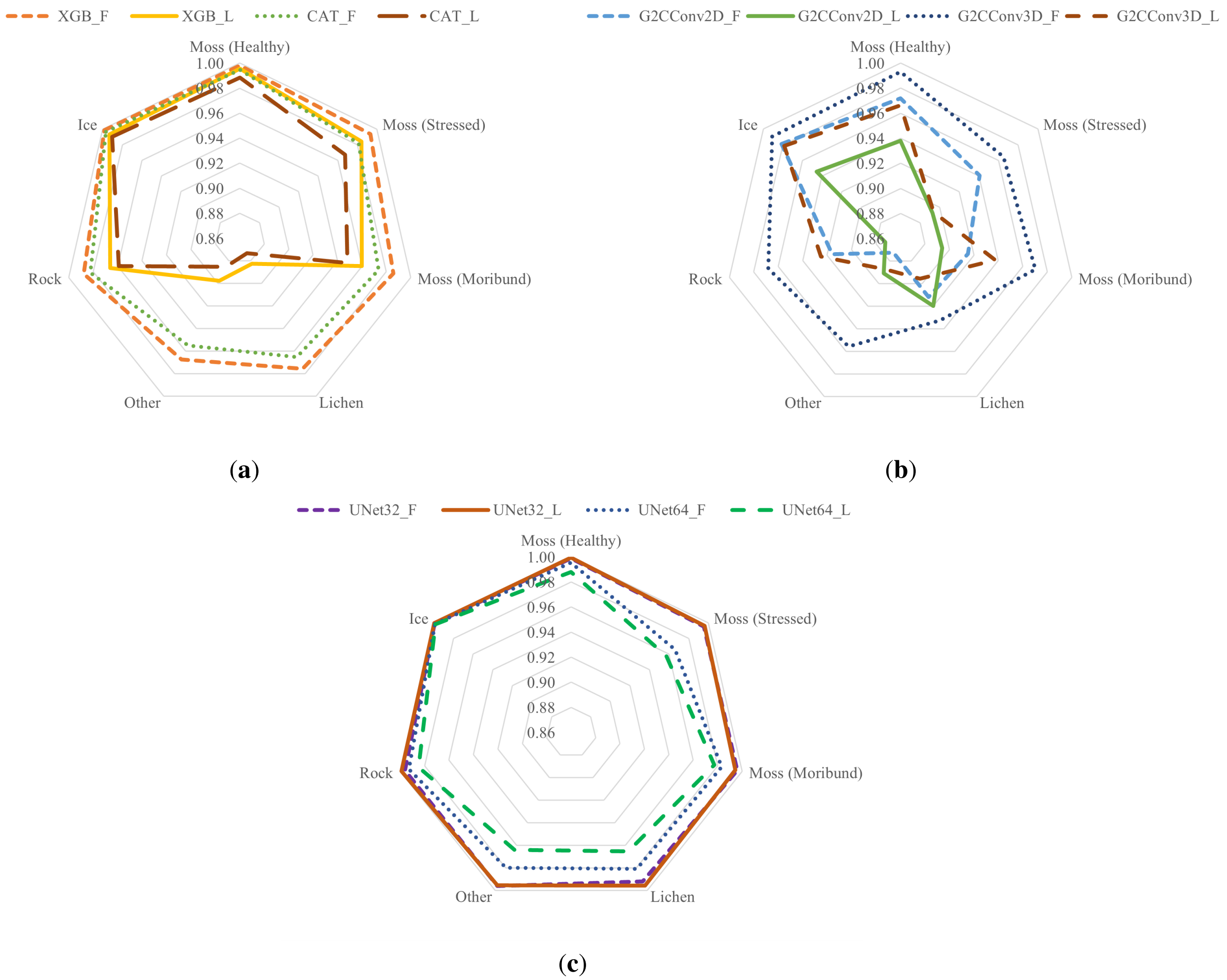

Figure S2. Visual comparison of F1-scores per class for the tested models, highlighting model performance variations across vegetation and material classes in ASPA 135. (**a**) Gradient boosting models (XGBoost and CatBoost) demonstrate strong performance across most classes, with particularly high F1-scores in "Moss (Healthy)" and "Ice," but show slight limitations in detecting the "Lichen" and "Other" classes. (**b**) G2C-Conv CNN models, including both 2D and 3D variants, exhibit broader generalisation with less fine-scale accuracy than gradient boosting models, especially in challenging classes like "Lichen" and "Other," where misclassification is more frequent. (**c**) UNet CNN models provide the best balance between generalisation and class-specific accuracy, particularly excelling in "Moss (Healthy)" and "Ice" classes, while effectively distinguishing between the majority of classes.

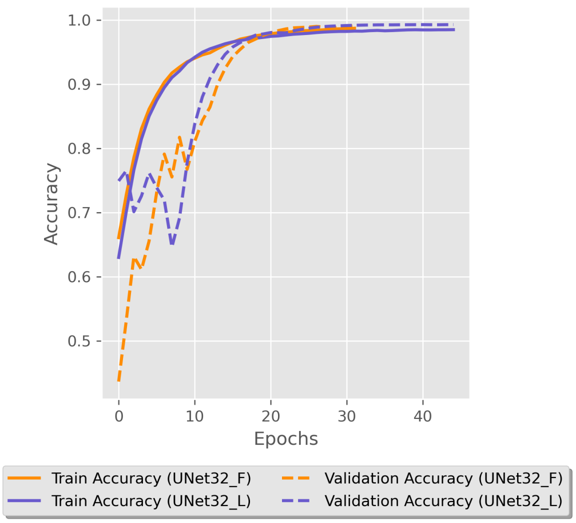

(**a**)

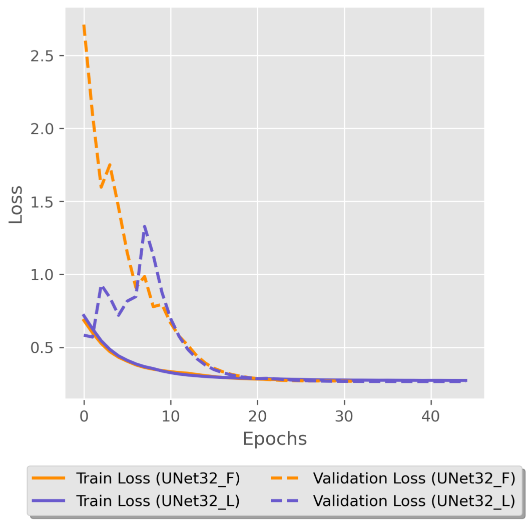

(**b**)

Figure S3. Training and validation curves for the UNet32 models across epochs. (**a**) Accuracy curves for the full (F) and light (L) models, showing consistent performance improvements throughout training. (**b**) Loss curves for the full and light models, illustrating the convergence trends over time.

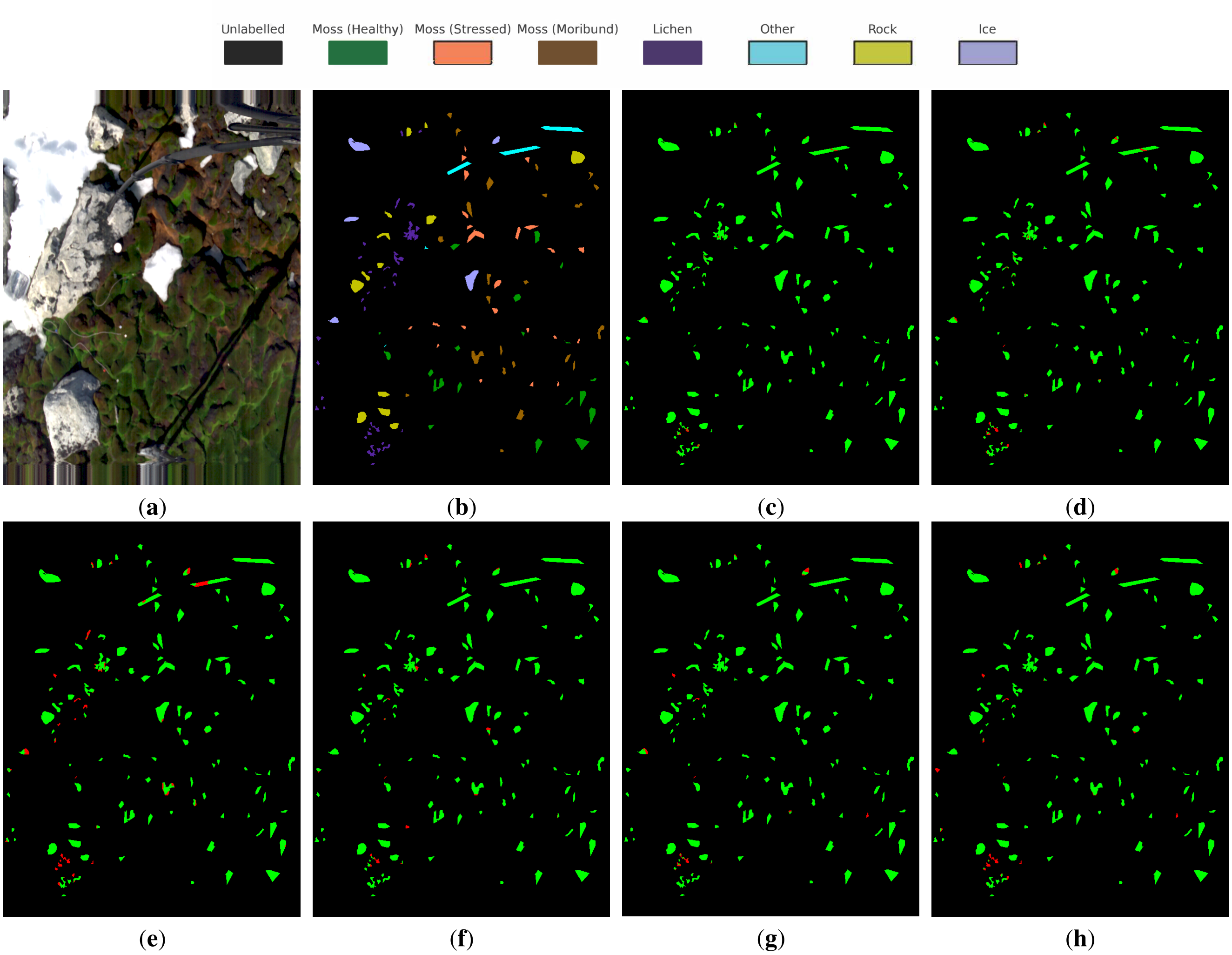

Figure S4. Comparison of model predictions from a labelled HSI scan for vegetation mapping at ASPA 135. (**a**) RGB colour composite of the HSI ground scan. (**b**) Ground truth label mask. (**c**) and (**d**) Predictions from XGB_F and CAT_F models. (**e**) and (**f**) Predictions from G2CConv2D_F and G2CConv3D_F models. (**g**) and (**h**) Predictions from UNet32_F and UNet64_F models. All predictions are masked to show only regions with ground truth labels for a fair visual comparison. Models were run using the full feature set; results from light input versions are not shown.

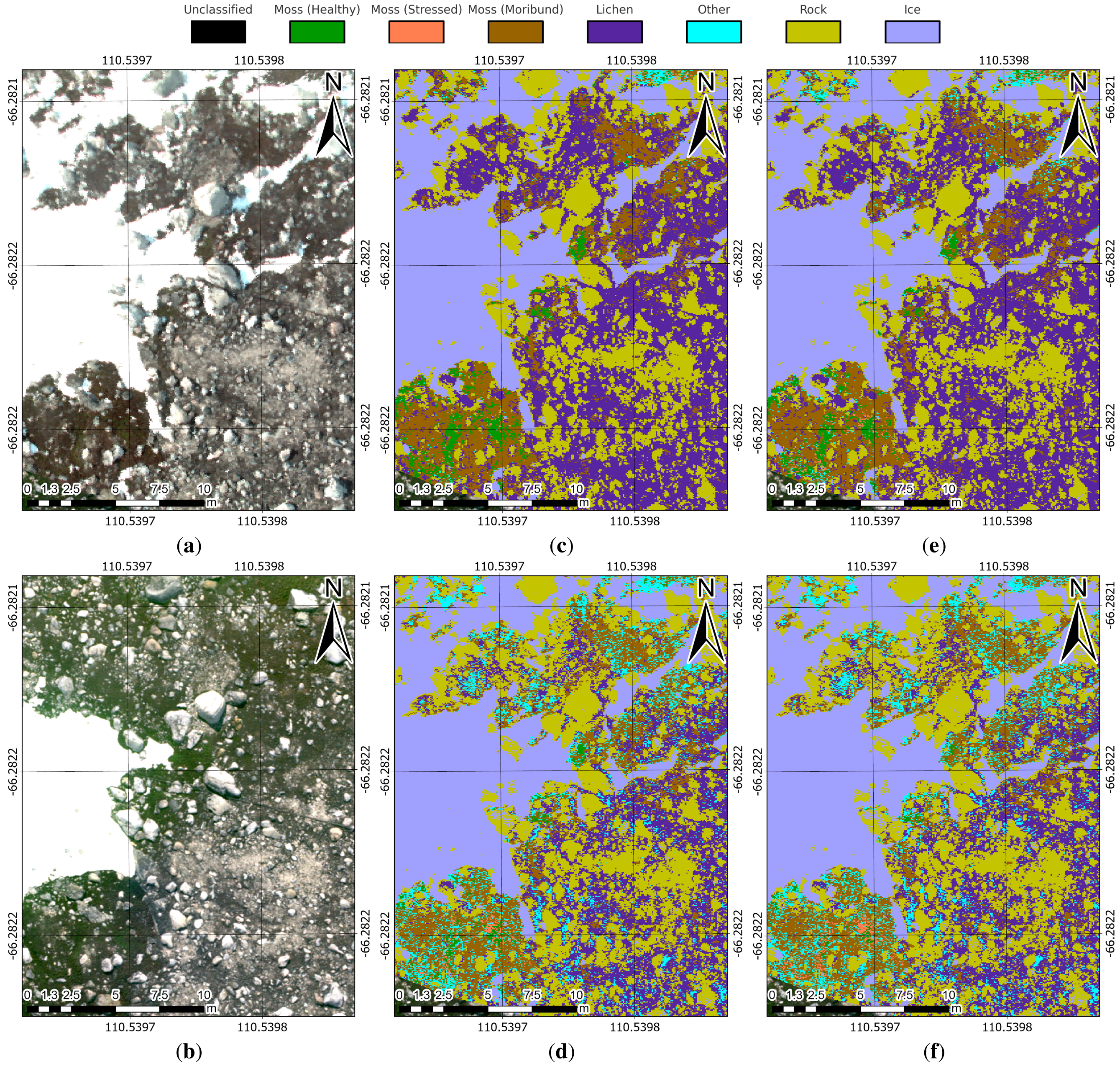

Figure S5. Predictions from gradient boosting models for mapping vegetation at ASPA 135. (**a**) HSI transect colour representation at 30 m AGL. (**b**) Aligned Sony RGB mosaic overlaid on the HSI map. (**c**) and (**d**) Predictions from XGB_F and XGB_L models. (**e**) and (**f**) Predictions from CAT_F and CAT_L models.

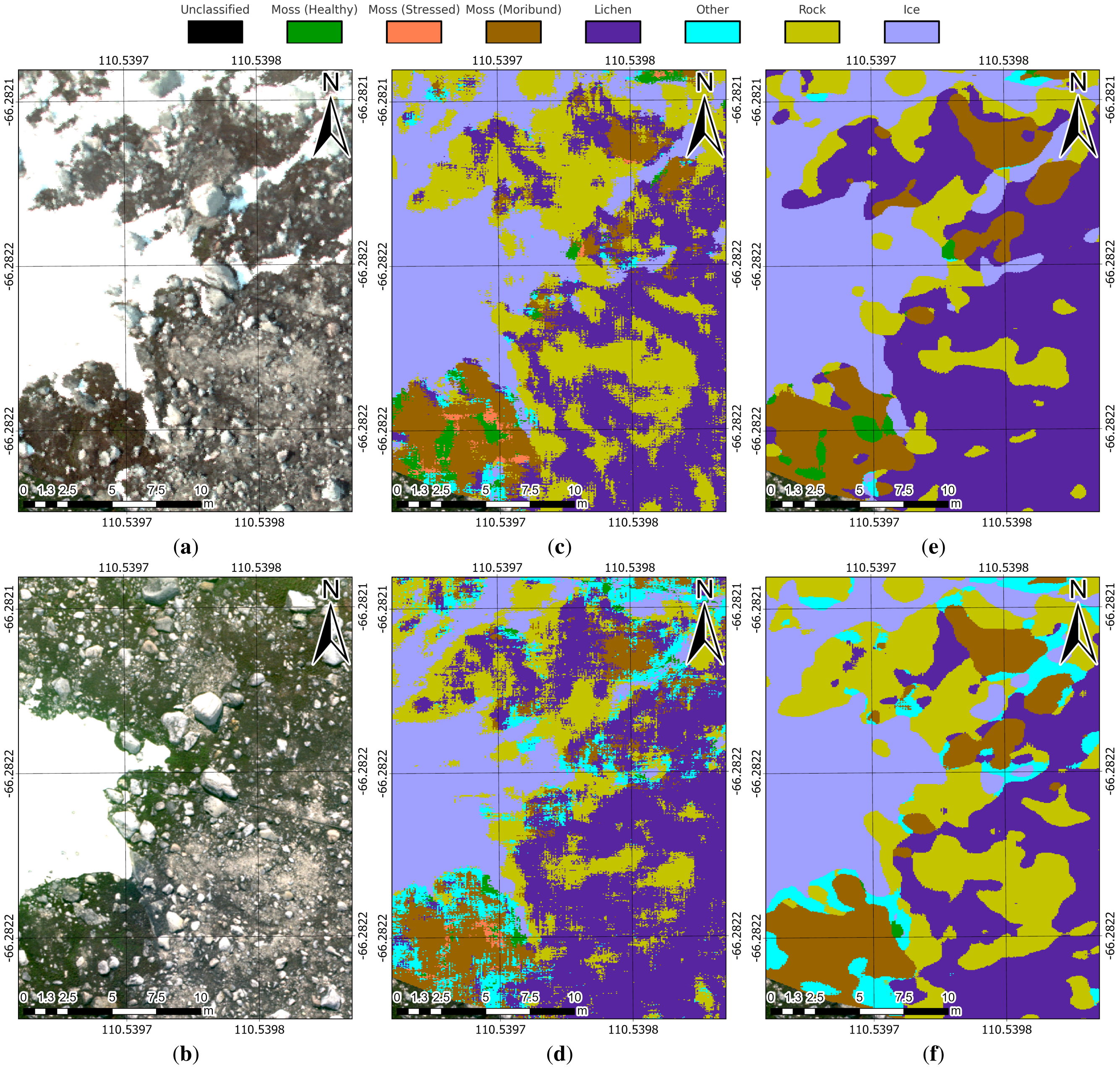

Figure S6. Predictions from G2C-Conv CNNs for mapping vegetation at ASPA 135. (**a**) HSI transect colour representation at 30 m AGL. (**b**) Aligned Sony RGB mosaic overlaid on the HSI map. (**c**) and (**d**) Predictions from G2CConv2D_F and G2CConv2D_L models. (**e**) and (**f**) Predictions from G2CConv3D_F and G2CConv3D_L models.

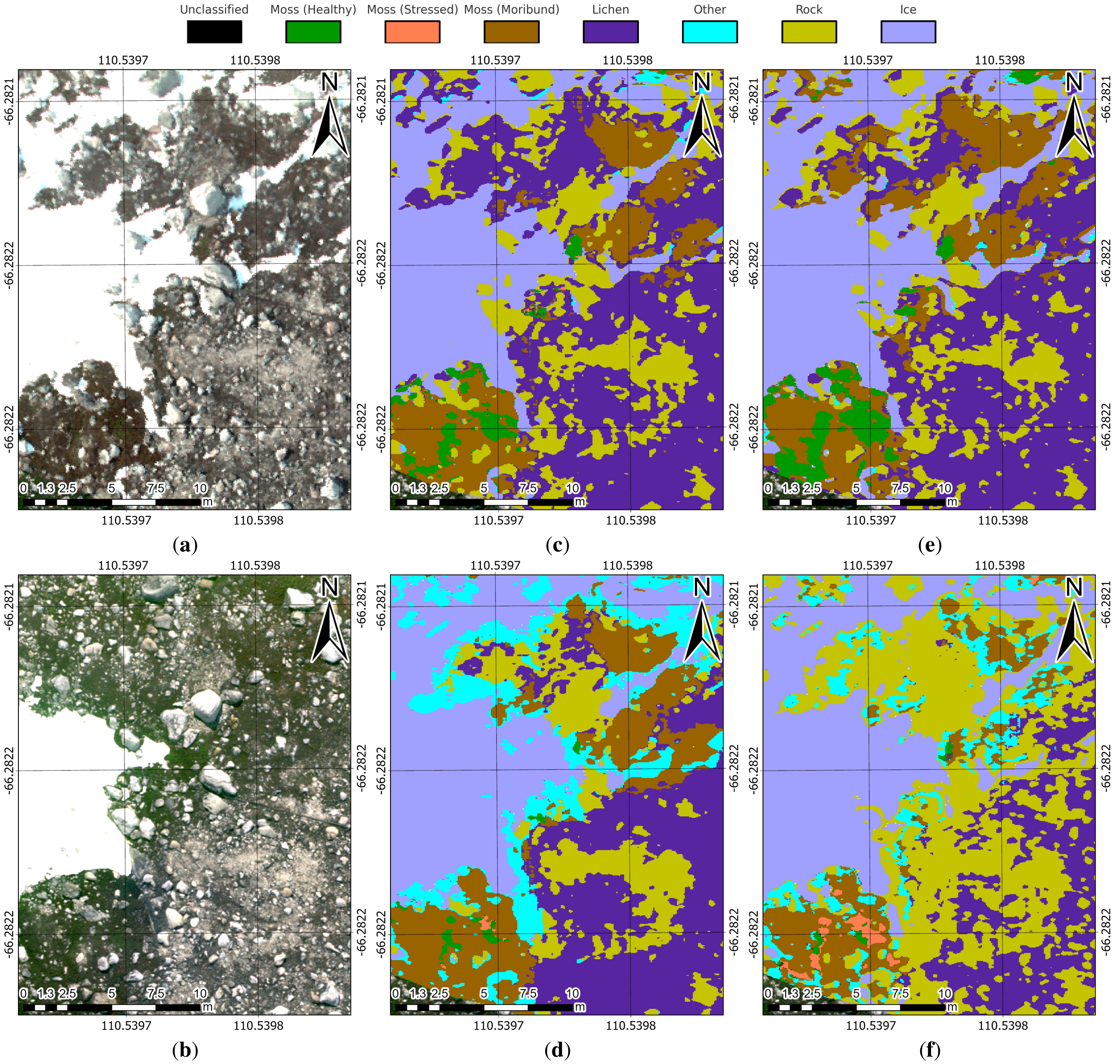

Figure S7. Predictions from UNet CNNs for mapping vegetation at ASPA 135. (**a**) HSI transect colour representation at 30 m AGL. (**b**) Aligned Sony RGB mosaic overlaid on the HSI map. (**c**) and (**d**): Predictions from UNet32_F and UNet32_L models. (**e**) and (**f**): Predictions from UNet64_F and UNet64_L models.

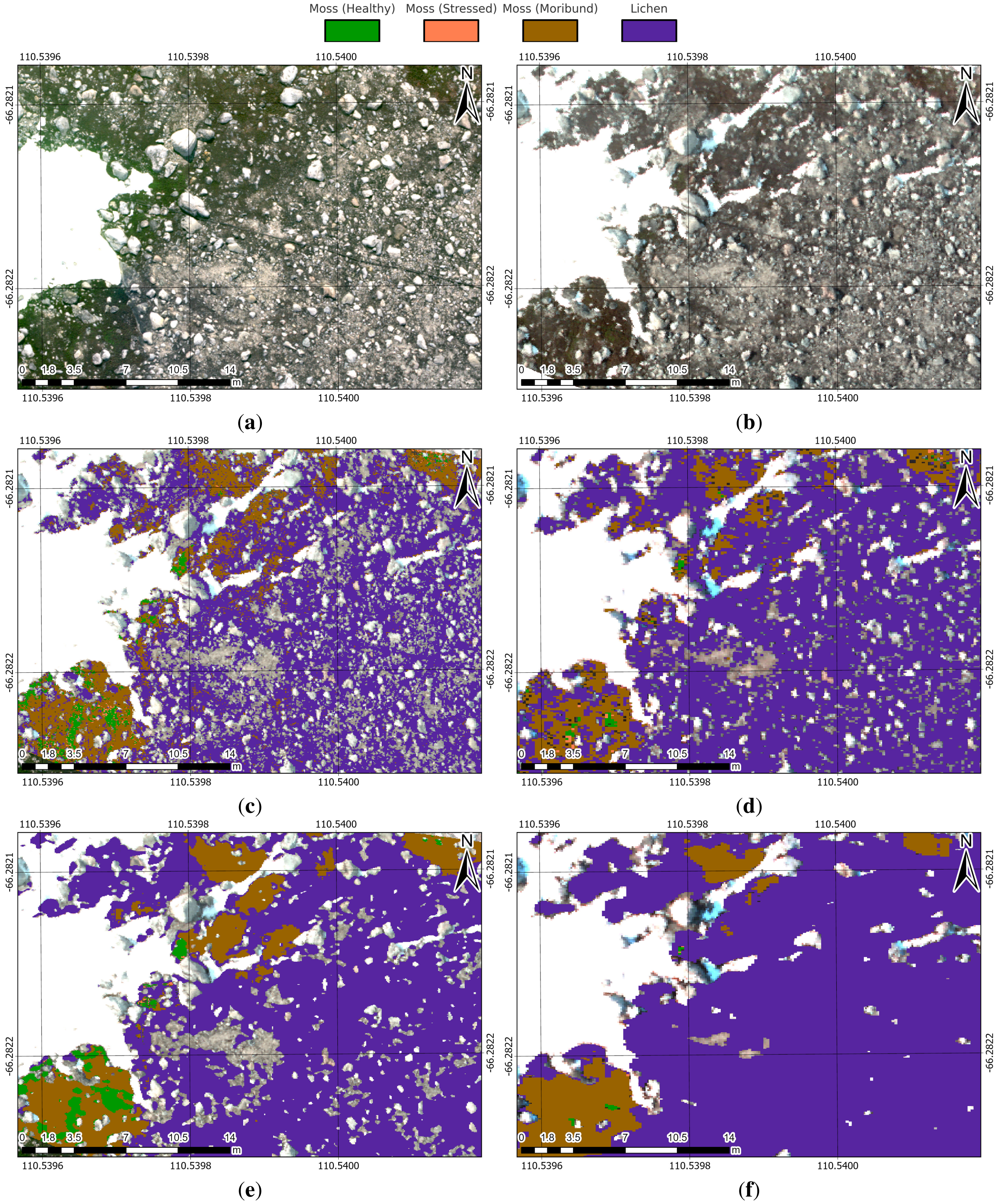

Figure S8. Vegetation mapping outputs from CAT_F and UNet32_F models at different altitudes. (**a**) RGB reference map. (**b**) Georeferenced HSI scan showing increased ice coverage due to later collection. (**c**) and (**d**): CAT_F predictions at 30 m and 70 m AGL, respectively. (**e**) and (**f**): UNet32_F predictions at 30 m and 70 m AGL, respectively. Higher-altitude maps show broader segmentation and more "Lichen" and "Moss (Moribund)," while lower-altitude maps capture finer detail in dense patches.

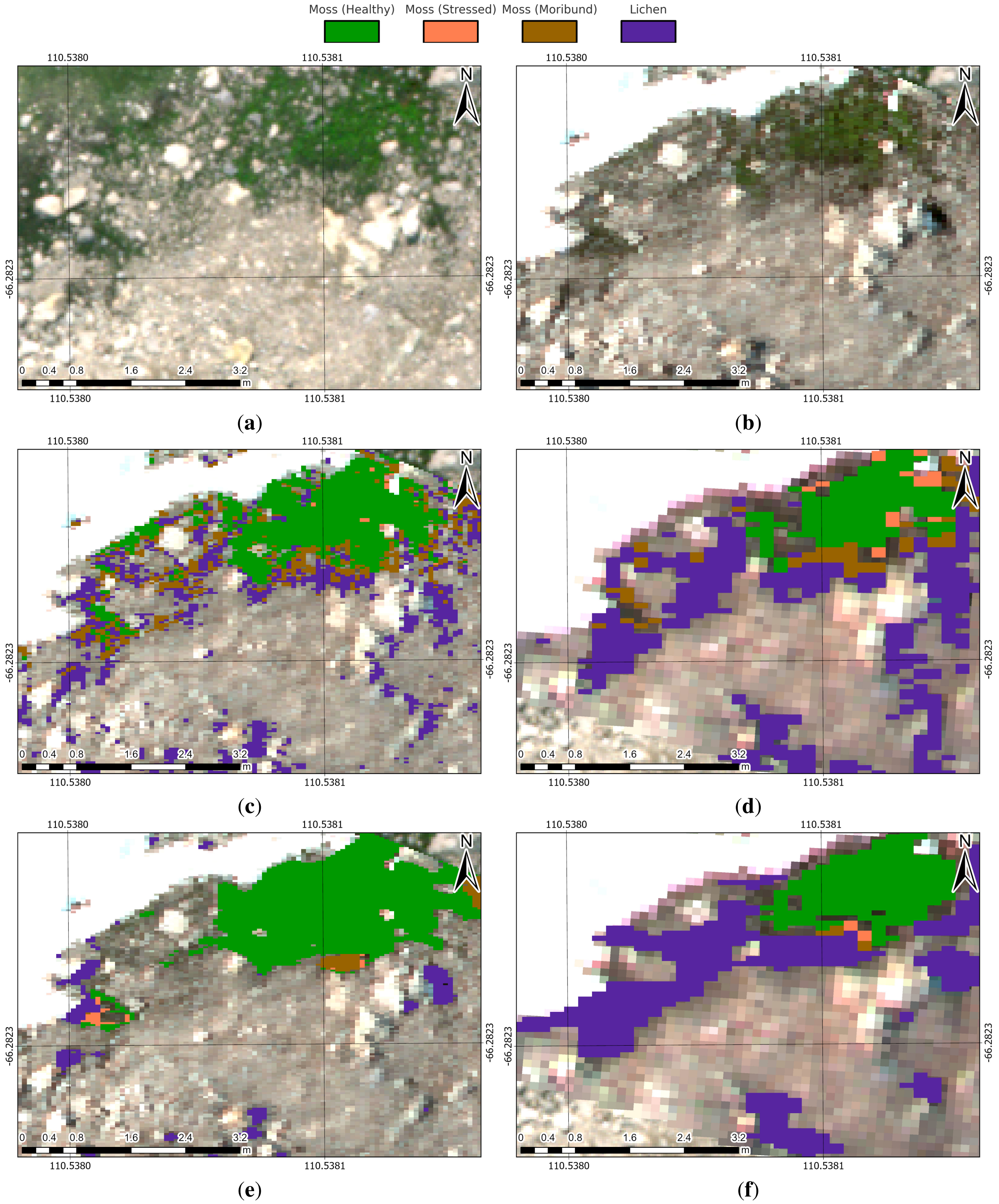

Figure S9. Comparison of vegetation classification at two HSI resolutions. (**a**) RGB map baseline for spatial reference. (**b**) Georeferenced HSI scan. (**c**) and (**d**): CAT_F segmentation at 30 m and 70 m AGL, respectively. (**e**) and (**f**): UNet32_F segmentation at 30 m and 70 m AGL, respectively. Higher resolution enables improved detection of dense vegetation classes in both datasets.

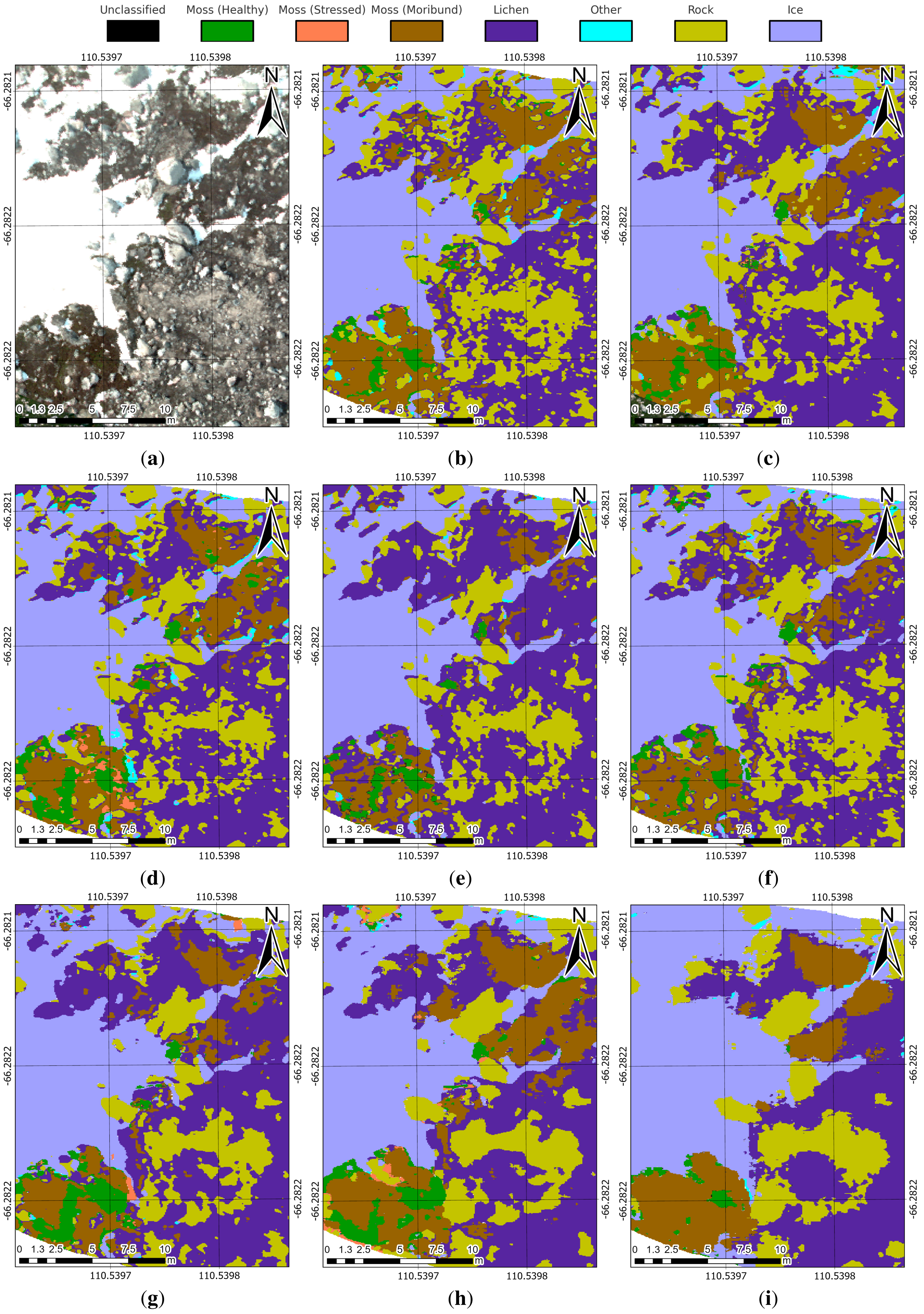

Figure S10. Comparison of UNet32_F vegetation segmentation with and without data augmentation. (**a**) RGB baseline. (**b**) No augmentation. (**c**) Proposed combination (brightness, noise, resize). (**d-i**) Individual augmentations: brightness, noise, resize, rotation, affine, and flip.
